# Melatonin–selenium nanoformulation: a promising therapeutic strategy against Ehrlich ascites carcinoma

**DOI:** 10.1038/s41598-026-53359-w

**Published:** 2026-05-26

**Authors:** Hanaa M. Morad, A. F. Abdel-Aziz, Mai M. Madkour

**Affiliations:** Department of Chemistry, Faculty of Science, Mansoura University, Mansoura, 35516 Egypt

**Keywords:** Melatonin-selenium nanoparticles, Ehrlich ascites carcinoma, Oxidative stress, Inflammation, Apoptosis, Cell cycle, Biochemistry, Cancer, Drug discovery

## Abstract

Combination therapy has emerged as a standard strategy for enhancing the efficacy of anticancer treatments. The purpose of our study was to assess the melatonin-selenium nanoparticles’ anticancer potential (MSeNPs) in a murine model of Ehrlich ascites carcinoma (EAC). Assessments included cell viability, hematological parameters, oxidative stress markers, apoptosis, cell cycle dynamics, and pro-inflammatory cytokines. Our findings demonstrate that MSeNPs inhibit tumor growth and enhance antioxidant defenses. MSeNPs significantly reduce IL-6 levels, alleviating EAC-associated inflammation. Furthermore, MSeNPs induced apoptosis through caspase-3 activation and Ki-67 downregulation, resulting in decreased cell proliferation and significant G0/G1 cell cycle arrest, accompanied by marked suppression of the S phase. In conclusion, these results highlight the synergistic therapeutic advantage of MSeNPs, indicating greater efficacy than monotherapies with melatonin, selenium, or selenium nanoparticles alone. MSeNPs hold promise as a potent, multi-targeted agent for future cancer therapies.

**Supplementary Information:**

The online version contains supplementary material available at 10.1038/s41598-026-53359-w.

## Introduction

It is well known that cancer is a potentially fatal disease characterized by abnormal and uncontrolled cell proliferation in any organ or tissue of the body. Treatments for cancer vary based on the type and stage of the disease and include clinical approaches such as surgery, radiotherapy, stem cell therapy, chemotherapy, immunotherapy, hormonal therapy, and targeted medications. Additionally, various products have shown promising potential in the prevention and treatment of cancer^1^. Despite this, the high cost of anticancer therapies and their detrimental adverse effects, along with the significant challenge of discovering effective drugs that can target various types of cancer, highlight the urgent need to develop new and more effective therapies^2^. The Ehrlich ascites carcinoma (EAC) mouse model is a well-known, rapidly proliferating, and highly reproducible transplantable tumor type that is frequently used for the preliminary evaluation of anticancer drugs. After intraperitoneal injection, the ascitic form develops. Tumor-induced inflammation and increased peritoneal vascular permeability led to ascites accumulation. This model is particularly useful for evaluating therapeutic efficacy, as it closely mimics aggressive tumor behavior^3^.

Nanotechnology, a promising new approach, has attracted increasing attention in cancer therapy research due to its potential to improve treatment outcomes^4^. Nanoparticles typically range in size from 10 to 100 nanometers and possess a large surface area, making them highly suitable for various biological applications. Due to their small size, nanomaterials can easily travel throughout the body, moving between organs and effectively penetrating targeted tissues. For both therapeutic and diagnostic applications, they can also be coupled with pharmacological molecules to target diseased tissues, such as cancer cells. Notably, nanoparticles are comparable in size to DNA and smaller than blood cells, which improves their capacity to interact at the molecular level^5^.

Melatonin (MEL), also known as N-acetyl-5-methoxytryptamine, is a naturally occurring hormone synthesized by various tissues in the human body. Although the pineal gland is the primary site of production, other tissues, including the bone marrow, retina, gastrointestinal tract, and lymphocytes, also synthesize it^6^. As a potent antioxidant, MEL effectively scavenges free radicals and inhibits oxidative stress in both in vitro and in vivo settings^7^. MEL is a lipophilic compound with a broad spectrum of biological anticancer effects, including notable anti-angiogenic properties^8^, as well as anti-migration, anti-invasion^9^, pro-apoptotic^10^, and anti-proliferative activities^11^. There is growing interest in the wide application of MEL for the treatment of various diseases, including inflammatory conditions, gastrointestinal disorders, cancer, mood disorders, and others^6^. MEL has demonstrated anticancer effects in several types of malignancies, including lung, cervical, gastric, breast, and colorectal cancers^12^. A possible interaction between micronutrient status and MEL-mediated biological effects is suggested by the important roles that trace elements like zinc, selenium, and magnesium play in enzymatic and antioxidant systems that are directly linked to MEL biosynthesis and activity^13^.

The trace element selenium (Se) is necessary for numerous biological functions. Specifically, at least 25 human selenoproteins, which are involved in a wide range of essential biological activities, include the amino acid selenocysteine, also known as the 21st amino acid. These include the regulation of reactive oxygen species (ROS), thyroid hormone metabolism, and immune function. Consequently, Se plays a critical role in modulating and preventing the clinical outcomes of various diseases, including cancer, diabetes, Alzheimer’s disease, mental health disorders, cardiovascular diseases, fertility issues, inflammatory conditions, and infections^14^. Se exists in both organic and inorganic forms; however, these forms exhibit limited absorption in the gastrointestinal tract and, more importantly, may exert toxic effects at high doses. These limitations have driven interest in the development of selenium nanoparticles (SeNPs), which aim to improve bioavailability and reduce toxicity, offering a safer and more efficient alternative for therapeutic and nutritional applications^15^.

Compared to traditional Se compounds, such as sodium selenate and sodium selenite, SeNPs demonstrate lower toxicity and greater biocompatibility, making them a safer and more effective option for therapeutic use^16^. Because of their distinct physicochemical characteristics, SeNPs have become an effective option for the clinical use of Se. These characteristics include high biocompatibility, enhanced stability, improved bioavailability, and reduced toxicity compared to conventional Se forms. These advantages position SeNPs as a valuable tool for advancing Se-based therapeutic strategies^17^.

Nanodrug delivery carriers offer the advantage of protecting incorporated therapeutic agents from degradation in physiological environments, enabling controlled and sustained release while reducing side effects and enhancing therapeutic efficacy. Se plays a critical role in MEL function by acting as a cofactor in enzymatic pathways that regulate antioxidant defenses, such as glutathione peroxidase and thioredoxin reductases, which functionally overlap with MEL’s antioxidative and cytoprotective roles. This functional overlap suggests a potential synergistic relationship. In this context, MEL-mediated protection against oxidative stress, inflammation, and cellular damage may be enhanced or complemented by Se. When paired with SeNPs, MEL not only improves the formulation’s bioavailability and stability but also enhances anticancer activity by inducing apoptosis, inhibiting cell cycle progression, and modulating oxidative stress^18^.

According to safety evaluations, SeNPs are more biocompatible and less cytotoxic than traditional Se compounds. The National Institutes of Health has established 400 µg of Se per day as the acceptable upper intake level for adults. A variety of dose descriptors, including NOAELs and LOAELs, for effects on body weight and other endpoints, are reported in a recent comprehensive study of repeated oral dose toxicity of SeNPs. In human investigations, a daily LOAEL of approximately 4.3 µg Se/kg body weight and a NOAEL of approximately 2.9 µg Se/kg body weight were observed. In animal models, NOAELs for body weight effects range from 0.24 to 1.2 mg Se/kg body weight per day; depending on the organ system evaluated, some endpoints exhibit even lower thresholds^19^. This collaborative approach offers an improved strategy for cancer treatment, particularly for tumor types that are aggressive or resistant^20^. According to reports, the addition of MEL improves antioxidant stability and reduces the likelihood of oxidative stress–mediated cytotoxicity, which is frequently associated with more reactive Se species. Preclinical studies suggest that MSeNPs may exhibit improved cellular compatibility compared to pure SeNPs, with lower hemolytic activity and reduced inflammatory responses in both in vitro and in vivo animal models^21^. In our study, we combined MEL with SeNPs for the treatment and pre-treatment of EAC, aiming to integrate the unique therapeutic properties of each agent. This combination strategy was designed to achieve enhanced outcomes and maximize therapeutic efficacy in combating cancer.

## Materials and methods

### Chemicals

The materials used were of pure chemical reagent grade and were used without further purification. Sodium selenite, selenious acid (98%), ascorbic acid (99%), and trypan blue dye were purchased from Sigma-Aldrich (USA). Carboxymethyl cellulose and MEL (≥ 99%) were purchased from Thermo Fisher Scientific (USA). Bovine serum albumin was purchased from Biowest. All other chemicals were of the highest analytical grade.

### Preparation of SeNPs

A total of 50 mL of double-distilled water was used to dissolve 0.2 g of sodium selenite and stirred thoroughly. Separately, 20 mL of double-distilled water was used to dissolve 0.2 g of bovine serum albumin under constant stirring. The two solutions were then combined and transferred into a clean autoclave, where the mixture was heated at approximately 120°C for 20 min, resulting in a reddish-coloured solution, showing that SeNPs are formed. After that, the mixture was centrifuged at 3000 rpm for 15 min. The supernatant was discarded, and the precipitate was washed with double-distilled water to remove any residual impurities. The purified SeNPs were then air-dried at room temperature and stored in a refrigerator^22^.

### Preparation of melatonin-selenium nanoparticles

**Fig. 1:**
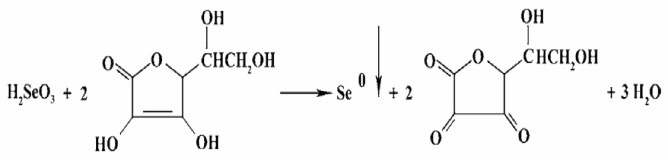
Schematic illustration of the formation of SeNPs via ascorbic acid–mediated reduction of selenious acid.

Melatonin–selenium nanoparticles (MSeNPs) were synthesized by reducing selenium acid with ascorbic acid while MEL was present. In this procedure, an appropriate amount of selenious acid (prepared as an aqueous solution of SeO₂) was thoroughly mixed with MEL. The selenious acid–MEL mixture was then supplemented with an excess of ascorbic acid to initiate the redox process. After 30 min of milling the reaction mixture, a light crimson product was produced, indicating the formation of MSeNPs (Fig. 1). For preservation, the product was kept at -20°C. The final formulation intended for medicinal applications consisted of a complex containing 95% MEL, 1% elemental Se, and 4% ascorbic acid. Prior to use, the product was dissolved in absolute ethanol and then briefly stabilized and suspended in a 0.5% carboxymethyl cellulose solution^20^.

### Characterization of SeNPs and MSeNPs

Characterization of SeNPs and MSeNPs was performed using various analytical techniques. The morphology, size, and structural properties of the nanoparticles were examined using a scanning electron microscope (SEM; JSM-6510 LV, JEOL, Japan) and a transmission electron microscope (TEM; JEM-100CX, JEOL, USA Inc., Peabody, MA). An X-Max 20 system was used to perform an energy-dispersive X-ray spectroscopy (EDX) investigation (Oxford Instruments, England). Particle size distribution and zeta potential were determined by dynamic light scattering using a Zetasizer Nano ZS (Malvern Instruments, Westborough, MA, USA).

### Analysis of combination index

The interaction between combined treatments was evaluated using the Combination Index (CI) method based on the Chou–Talalay equation. CI was calculated according to the following formula:

$$\:\mathrm{C}\mathrm{I}=\frac{\left({\mathrm{D}}_{1}\right)}{{\left(\mathrm{D}\mathrm{x}\right)}_{1}}+\frac{\left({\mathrm{D}}_{2}\right)}{{\left(\mathrm{D}\mathrm{x}\right)}_{2}}$$

where $\:{\mathrm{D}}_{1}$ and $\:{\mathrm{D}}_{2}$ are the doses of MEL and Se in combination required to achieve a given effect level, and $\:{\left(\mathrm{D}\mathrm{x}\right)}_{1}$ and $\:{\left(\mathrm{D}\mathrm{x}\right)}_{2}$are the doses of the same agents required to produce the same effect when used individually. CI values were interpreted as follows: CI < 1 indicates synergism, CI = 1 indicates an additive effect, and CI > 1 indicates antagonism.

### Determination of encapsulation efficiency

 Calibration curves of MEL were constructed using a stock solution (100 µg/mL) prepared in ethanol. Standard solutions in the concentration range of 5–100 µg/mL were obtained by serial dilution of the stock solution with the same solvent. A quartz cuvette-equipped UV-Vis spectrophotometer was used to test each standard solution’s absorbance at 278 nm (T80 + UV/VIS Spectrometer). Following nanoparticle preparation, the dispersion was centrifuged at high speed to separate nanoparticles from the unencapsulated drug. The resulting supernatant, containing free MEL, was carefully collected and diluted (1:20,000) with ethanol to confirm that the concentration was within the calibration curve’s linear range. The absorbance of the diluted samples was measured at 278 nm^23^. The concentration of free MEL was calculated from the calibration curve after correction for the dilution factor. The following formula was used to calculate the encapsulation efficiency (EE%):

$$\:\mathrm{E}\mathrm{E}\:\%=\frac{\mathrm{T}\mathrm{o}\mathrm{t}\mathrm{a}\mathrm{l}\ \mathrm{D}\mathrm{r}\mathrm{u}\mathrm{g}-\mathrm{F}\mathrm{r}\mathrm{e}\mathrm{e}\ \mathrm{D}\mathrm{r}\mathrm{u}\mathrm{g}}{\mathrm{T}\mathrm{o}\mathrm{t}\mathrm{a}\mathrm{l}\ \mathrm{D}\mathrm{r}\mathrm{u}\mathrm{g}}\:\times\:100$$

### Acute toxicity study

The mice were divided into four equal groups (six per group). MSeNPs were administered orally as a single dose of 2, 5, 10, and 20 mg/kg body weight to each group. For twenty-four hours, the animals were monitored for any adverse changes in behavior, while morbidity and mortality were assessed at least twice a day.

### Ehrlich ascites carcinoma cells

The EAC cell line was used in the present study. This cell line was obtained from the National Cancer Institute, Cairo University, Egypt. The tumor line was maintained through serial intraperitoneal (i.p.) transplantation of 1 × 10^6^ tumor cells suspended in 1 mL of saline into mice using a 25G needle. Mice inoculated with tumor cells intraperitoneally typically show detectable tumor effects 3–5 days post-injection. The EAC cells exhibited rapid proliferative capacity, causing ascitic fluid to gradually accumulate and eventual mortality of the host animals within approximately three weeks^24,25^.

### Experimental animals and tumor induction

All experiments in this study were conducted on adult female Swiss albino mice weighing between 20 and 26 g, obtained from Abu Rawash, Giza, Egypt. The animals were housed individually in standard laboratory cages under controlled environmental conditions, with free access to food and water. Prior to the start of the experiments, the mice were acclimatized for one week under the same laboratory conditions. At the end of the investigation, the mice were anesthetized and euthanized *via* isoflurane inhalation, in accordance with institutional ethical approval from the Faculty of Science, Mansoura University, Egypt. All procedures were performed in compliance with the ARRIVE guidelines.

A total of 66 female Swiss albino mice were used in this study. The animals were initially divided into two main groups: pre-treatment and treatment groups. In the pre-treatment groups, the tested compounds were administered orally in three separate doses per animal prior to tumor induction. Subsequently, all mice (in both pre-treatment and treatment groups) were intraperitoneally administered a 1 mL EAC cell suspension injection to induce tumor formation. The day of tumor implantation was designated as day 0. On the following day (day 1), the animals were randomly divided into 11 groups, each consisting of six mice. The tested compounds were then administered orally every other day for two weeks (six doses in total), starting after tumor induction. The mice were sacrificed for additional experimental investigation two days following the last dosage.

### Experimental design

Group 1 (Negative Control): Healthy mice received an oral administration of 0.2 mL isotonic saline solution and served as the negative control group. Group 2 (Untreated Control): EAC-bearing mice received no treatment until the day of sacrifice and served as the untreated control group. Group 3 (Se Treatment): EAC-bearing mice were orally administered sodium selenite (0.1 mg/kg b.w.). Group 4 (Se Pre-treatment): Mice were pretreated orally with sodium selenite (0.1 mg/kg b.w.), followed by EAC induction. Post-induction, sodium selenite was continued at the same dose. Group 5 (MEL Treatment): EAC-bearing mice were orally administered MEL (10 mg/kg b.w.). Group 6 (MEL Pre-treatment): Mice were pretreated orally with MEL (10 mg/kg b.w.), followed by EAC induction. Post-induction, MEL was continued at the same dose. Group 7 (SeNPs Treatment): EAC-bearing mice were orally administered SeNPs (10 mg/kg b.w.). Group 8 (SeNPs Pre-treatment): Mice were pretreated orally with SeNPs (10 mg/kg b.w.), followed by EAC induction. Post-induction, SeNPs were continued at the same dose. Group 9 (MSeNPs Treatment, Low Dose): EAC-bearing mice were orally administered MSeNPs (5 mg/kg b.w.). Group 10 (MSeNPs Treatment, High Dose): EAC-bearing mice were orally administered MSeNPs (10 mg/kg b.w.). Group 11 (MSeNPs Pre-treatment): Mice were pretreated orally with MSeNPs (10 mg/kg b.w.), followed by EAC induction. Post-induction, MSeNPs were continued at the same dose.

### Ascitic tumor collection and tissue sample processing

Ascitic fluid was collected post-mortem from the peritoneal cavity using a sterile syringe. Samples were collected separately from each experimental group and centrifuged at 1800 rpm for 5 min using a BOOKMAN GS-6R centrifuge with a rotor radius of 17 cm. The resulting pellets were then separately homogenized in phosphate-buffered saline (PBS, pH 7.4) at a ratio of 5 mL per gram of cells. The homogenates were further centrifuged at 4000 rpm for 15 min at 4°C. The resulting supernatants were collected and stored at − 80°C until further analysis. After excision, the liver and kidneys were dissected, rinsed with cold isotonic saline, and blotted between two pieces of filter paper. The kidneys were preserved in 10% formalin for subsequent histopathological analysis. An accurately weighed liver tissue sample was homogenized in phosphate-buffered saline (PBS, pH 7.4) using a tissue homogenizer. The homogenate was then diluted to prepare a 10% (w/v) liver homogenate. Cell debris and nuclei were removed by centrifuging the samples at 12,000 × g for 20 min at 4°C.

### Estimation of hematological parameters

Blood samples were collected and analyzed to evaluate various hematological parameters, including hemoglobin (Hb) concentration, red blood cell (RBC) count, and white blood cell (WBC) count, using standard laboratory procedures. Additional parameters assessed included hematocrit (%), mean corpuscular volume (fL), mean corpuscular hemoglobin (pg), platelet count (×10³/µL), and the percentages of neutrophils and lymphocytes.

### Tumor volume and evaluation of viability

On the 14th day post-inoculation, tumor progression was further evaluated by measuring the volume of ascitic fluid accumulated in the peritoneal cavity of experimental mice. The Trypan Blue exclusion assay was performed to evaluate EAC cell viability, where viable cells excluded the dye and appeared unstained, whereas non-viable cells took up the dye and appeared blue under microscopic observation^26^.

### Biochemical assessment of oxidative stress parameters

Oxidative stress levels were evaluated by monitoring the activity of significant antioxidant enzymes and related biomarkers. Catalase (CAT) activity was assessed by incubating the sample with substrate at 37°C for 3 min. The reaction was stopped using ammonium molybdate, and the absorbance of the resulting yellow complex formed between molybdate and hydrogen peroxide was measured at 374 nm against a blank^27^. Superoxide dismutase (SOD) activity was measured according to the method of Nishikimi et al.^28^. Glutathione peroxidase (GPx) activity was determined using a commercial kit (Bio-Diagnostic Company, Cat.No. GP 2524)^29^. Lipid peroxidation was evaluated by measuring malondialdehyde (MDA) levels using the method described by Ohkawa et al.^30^. Additionally, nitric oxide (NO) levels were determined via the Griess assay using the supernatant of ascitic fluid^31^.

### ELISA assay

Interleukin-6 (IL-6) levels were measured using the Elabscience^®^ Mouse IL-6 ELISA Kit (Cat. No. E-EL-M0044) on the supernatant obtained from washed ascitic fluid.

### Analysis of cell cycle

EAC cells were chilled on ice and then fixed with 70% ethanol, followed by washing with cold phosphate-buffered saline. The cells were then resuspended in propidium iodide solution. Flow cytometric analysis was performed using a BD FACSCalibur™ flow cytometer (Becton Dickinson, Mountain View, CA, USA).

### Analysis of caspase-3 by flowcytometry

Caspase-3 levels were detected using direct immunofluorescence with the paraformaldehyde/saponin method. The assay was performed using the Bio-Plex Pro™ RBM Apoptosis Panel 3 (Cat. No. 171WAR3CK) according to the manufacturer’s instructions.

### Immunohistochemical study

Ki-67 expression was assessed using the Ki-67 (SP6) Rabbit Monoclonal Antibody kit (Sigma-Aldrich, Cat. No. 275R-17). Formalin-fixed, paraffin-embedded tumor tissue sections (~ 3 μm thick) were mounted on positively charged slides, baked at 60°C, then deparaffinized and rehydrated. Antigen retrieval was performed using heat-induced epitope retrieval with Trilogy™ solution for 30 min. Slides were washed three times with phosphate-buffered saline and blocked with 5% bovine serum albumin for 60 min at room temperature. The primary antibody was diluted 1:100–1:500 using an appropriate antibody diluent and incubated at room temperature for 30 min. Immunodetection was performed using a horseradish peroxidase polymer-based detection system with amplification for 10 min, followed by chromogen development with diaminobenzidine for 2–5 min. Slides were counterstained with Mayer’s hematoxylin for 13 min, dehydrated, and coverslipped for microscopic examination^32^.

### Histopathological analysis

Kidney tissue samples from the various experimental groups were collected, rinsed with saline solution, and sectioned into small pieces. These tissue sections, along with EAC cells, were promptly fixed in 10% formalin for 24 h. After fixation, the samples were dehydrated through a graded series of ethanol concentrations, ending with two changes in absolute ethanol (100%). The tissues were then cleared with xylene prior to paraffin embedding. The paraffin-embedded samples were stained with hematoxylin and eosin (H&E) after being sectioned at a thickness of 4–5 μm. An electric light microscope (Olympus CX41, Japan) was used for the histopathological evaluation. Blinded histopathological assessment was performed to minimize observer bias. Tissue changes were graded using a semi-quantitative ordinal scale: absent (−), mild (+), moderate (++), and severe (+++), and interobserver agreement was evaluated using the weighted Cohen’s kappa coefficient.

### Statistical analysis

Data are presented as mean ± standard error (SE). Each experiment was performed in at least three independent replicates. Statistical analyses were conducted using ANOVA and descriptive statistics in GraphPad Prism (version 8). A p-value < 0.05 was considered statistically significant.

## Results

### Characterization of SeNPs

**Fig. 2:**
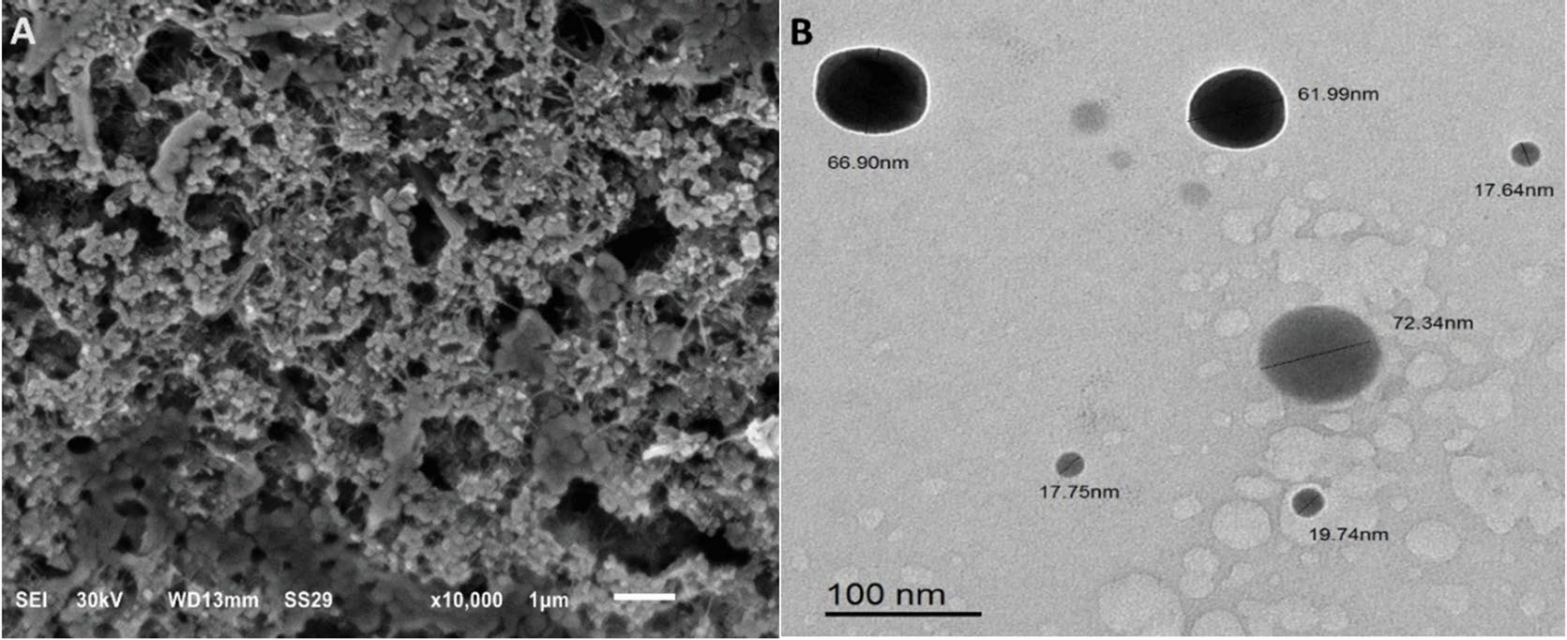
(**A**) SEM image of SeNPs with different particle sizes (62–192 nm). (**B**) TEM images of SeNPs showing various particle sizes and morphologies, with a scale bar = 100 nm.

**Fig. 3:**
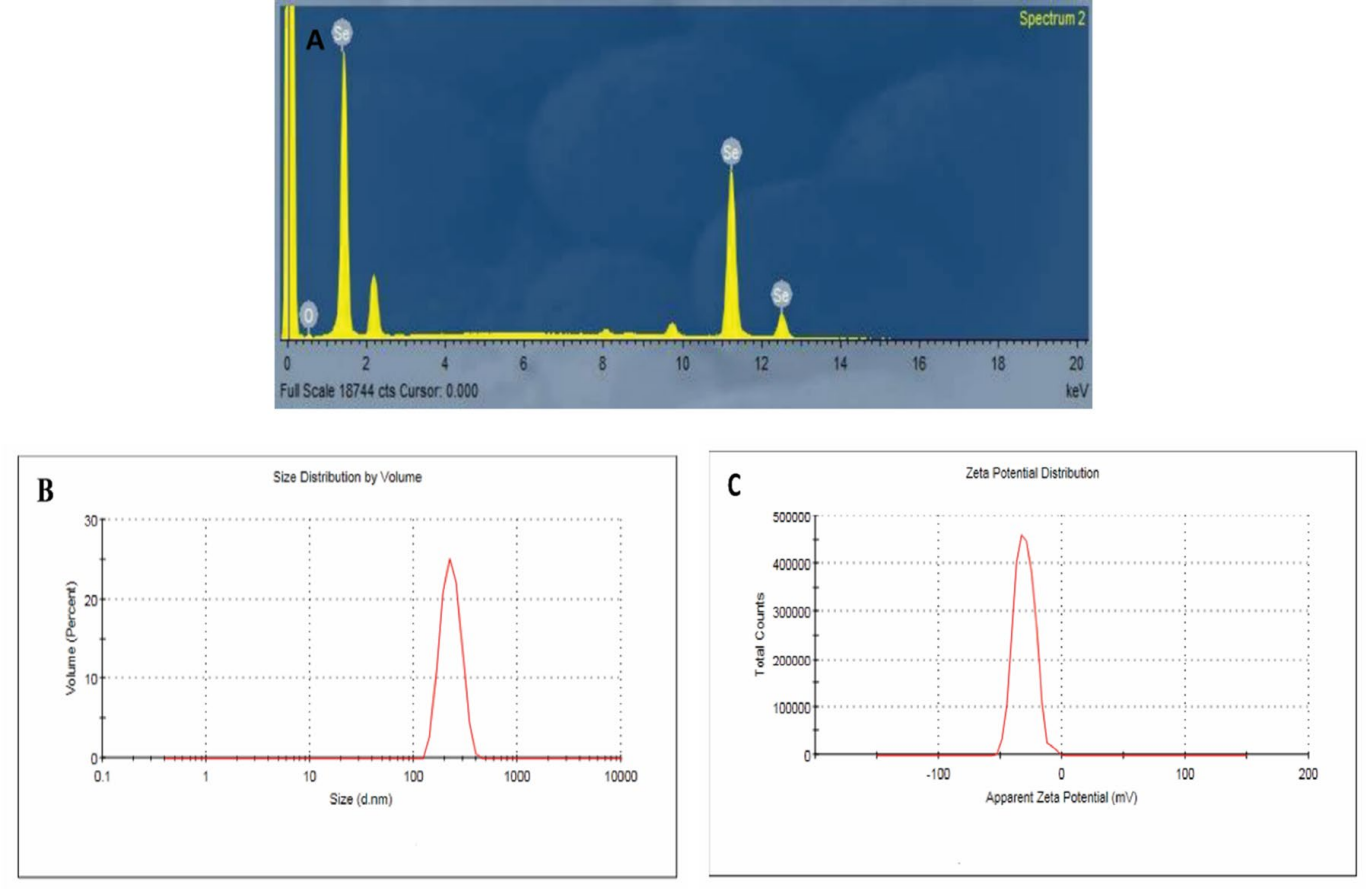
Characterization of SeNPs: (**A**) EDX analysis, (**B**) particle size distribution, and (**C**) zeta potential distribution.

The synthesized SeNPs were spherical, exhibited a broad size distribution, and demonstrated good colloidal stability, making them suitable for applications such as drug delivery and sensing. SEM analysis revealed particle sizes ranging from 62 to 192 nm (Fig. 2A), while TEM images provided detailed views of particle sizes and morphologies at a scale of 100 nm (Fig. 2B). EDX analysis confirmed the elemental composition of the nanoparticles, verifying the presence of Se (Fig. 3A). The average particle size determined by dynamic light scattering was 230 ± 49.9 nm, slightly larger than the SEM measurements, possibly due to differences in measurement techniques or particle aggregation (Fig. 3B). The zeta potential was measured at − 30.5 ± 8.19 mV, indicating good colloidal stability due to electrostatic repulsion between particles (Fig. 3C).

#### Characterization of MSeNPs

**Fig. 4:**
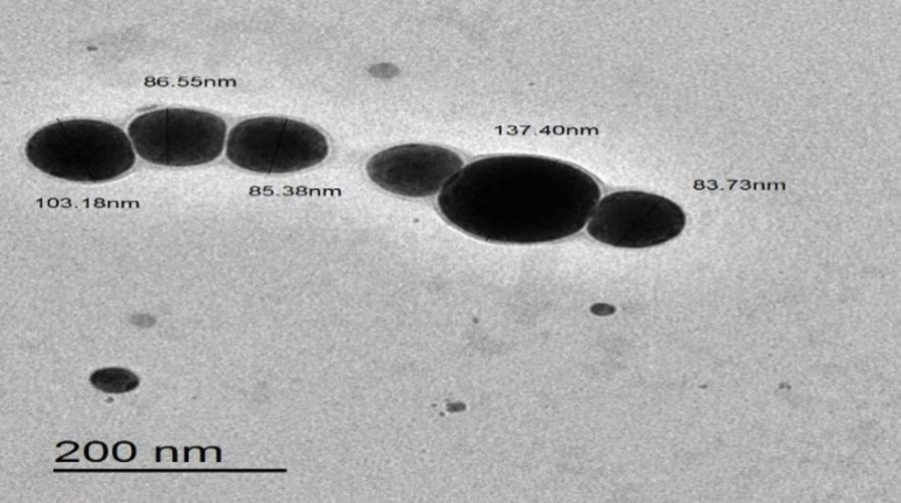
TEM images of MSeNPs showing different particle sizes and morphology, with a scale bar = 200 nm.

**Fig. 5:**
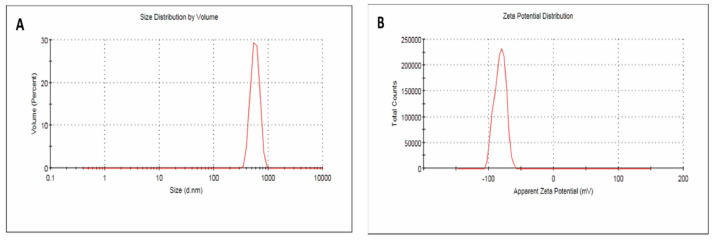
Characterization of MSeNPs: (**A**) particle size distribution, (**B**) zeta potential distribution.

MSeNPs exhibited varied particle sizes and morphologies, as shown at a 200 nm scale bar (Fig. 4), providing detailed structural insights. The nanoparticles retained a predominantly spherical shape, with an average particle size of 574.4 ± 102.2 nm, significantly larger than that of SeNPs. This size increase may result from the incorporation of MEL or from particle agglomeration. The zeta potential was measured at − 82.3 ± 8.53 mV, indicating excellent colloidal stability due to strong electrostatic repulsion that minimizes aggregation (Fig. 5A, B). Overall, the MSeNPs demonstrated spherical morphology, larger particle size, and very high colloidal stability, making them promising candidates for applications that require stable and functional nanoparticles.

### Combination index of MSeNPs

Two combination doses (5 mg/kg and 10 mg/kg) were analyzed based on their proportional composition (MEL: 95%, Se: 1%). For the 5 mg/kg combination, the calculated doses were 4.75 mg/kg MEL and 0.05 mg/kg Se. The resulting CI value was 0.48, indicating a synergistic interaction (CI < 1). For the 10 mg/kg combination, the calculated doses were 9.5 mg/kg MEL and 0.1 mg/kg Se. The CI value was 0.96, indicating an approximately additive effect with slight synergism. Overall, these findings demonstrate that the MSeNPs exhibit dose-dependent interaction, with stronger synergy observed at the lower dose level.

### Encapsulation efficiency of MSeNPs

The calibration curve of MEL exhibited good linearity within the concentration range of 5–40 µg/mL; however, at higher concentrations, a plateau in absorbance was observed, indicating deviation from linearity. The absorbance of the diluted supernatant was within the linear range, enabling accurate quantification of free MEL. Following correction for the dilution factor, the encapsulation efficiency (EE%) of the nanoparticles was determined to be approximately 69%.

### Acute toxicity study

Compared with safety data, the acute toxicity test at the limit doses of 2, 5, 10, and 20 mg/kg body weight of MSeNPs caused no changes in mice behavior and no lethality during the 15 days of observation. The compound may therefore be safe at these dosages, as the LD50 in mice was higher than 20 mg/kg body weight. For this study’s in vivo investigation, dosages of 5 and 10 mg/kg were employed.

### Effect of MSeNPs on tumor volume in EAC-bearing mice in different groups

**Fig. 6:**
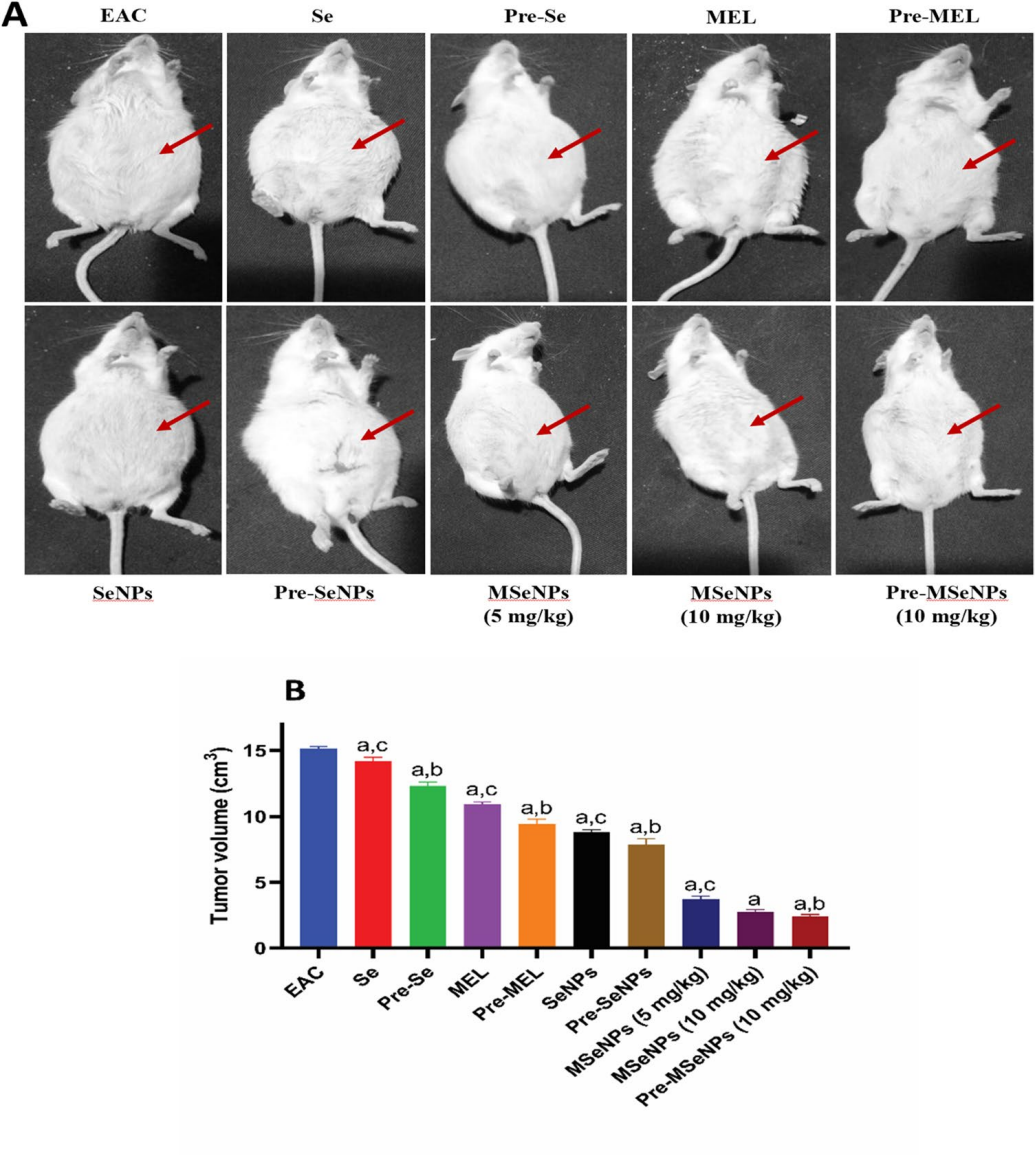
(**A**) Representative photos of tumor-bearing mice with various treatments. (**B**) Effect of MSeNPs on tumor volume in EAC-bearing mice in different groups. EAC: Ehrlich Ascites Carcinoma; Se: treatment of selenium; Pre-Se: pre-treatment of selenium; MEL: treatment of melatonin; Pre-MEL: pre-treatment of melatonin; SeNPs: treatment of selenium nanoparticles; Pre-SeNPs: pre-treatment of selenium nanoparticles; MSeNPs: melatonin-selenium nanoparticles; Pre-MSeNPs: pre-treatment of melatonin-selenium nanoparticles. Different letters indicate significant differences at *p* < 0.0001. a: all groups vs. EAC-bearing mice control group; b: Pre-treatment groups vs. their corresponding treatment groups; c: treatment groups vs. MSeNPs at 10 mg/kg. Data are presented as mean ± SE.

EAC cell growth in mice under different treatments was monitored. Representative images were taken, and at the end of the experiment, tumor volumes were measured (Fig. 6A, B). Tumor volume was significantly greater in the untreated EAC-bearing mice compared to all treatment groups (*p* < 0.0001). Administration of SeNPs caused the tumor volume to significantly decrease relative to sodium selenite (*p* < 0.0001). Furthermore, treatment with MSeNPs at a dosage of 10 mg/kg produced a significantly greater reduction in the volume of the tumor compared to sodium selenite (*p* < 0.0001), SeNPs (*p* < 0.0001), and MEL (*p* < 0.0001). Similarly, MSeNPs at a dose of 5 mg/kg led to significantly smaller tumor volumes than those observed in the sodium selenite (*p* < 0.0001), SeNPs (*p* < 0.0001), and MEL (*p* < 0.0001) groups. However, there was no statistically significant difference between the MSeNPs treatment groups receiving 5 mg/kg and 10 mg/kg.

Tumor volumes in the pre-treatment groups were smaller than those in the corresponding treatment groups. Pre-treatment with MSeNPs at 10 mg/kg resulted in the lowest tumor volume compared to MSeNPs treatment at 5 mg/kg (*p* = 0.0445) and 10 mg/kg (*p* = 0.9955). Pre-treatment with MEL was also more effective than MEL treatment (*p* = 0.0159), while pre-treatment with sodium selenite was significantly more effective than its treatment counterpart (*p* = 0.0019). Similarly, pre-treatment with SeNPs resulted in smaller tumor volumes than SeNP treatment, although this difference was not statistically significant (*p* = 0.3071). Overall, MSeNPs demonstrated the strongest cytotoxic effect on EAC cells, particularly when administered as a pre-treatment at 10 mg/kg. These results are summarized in Fig. 6B.

### Estimation of hematological parameters

The EAC group exhibited severe hematological disturbances, including markedly reduced Hb, RBC counts, and platelet counts, along with significantly elevated WBC counts and an imbalanced immune cell profile (increased neutrophils and decreased lymphocytes), reflecting the impact of tumor progression. Treatment with either sodium selenite or MEL alone produced moderate improvements in these parameters. Pre-treatment with either agent further enhanced their protective effects, particularly in restoring RBC counts, Hb levels, and immune cell balance. Administration of SeNPs significantly improved all hematological parameters and corrected the immune cell ratio. Pre-treatment with SeNPs also showed beneficial effects, although slightly less pronounced than post-treatment. MSeNPs produced the most pronounced improvements, restoring Hb, RBC, and platelet counts and achieving the highest lymphocyte percentage, indicating strong hematological recovery. Overall, pre-treatment enhanced outcomes across all groups. These results are summarized in Table 1.

**Table 1:** Effect of MSeNPs on hematolgical parameters in EAC-bearing mice in different groups.

Parameter	Groups										
	Units	EAC	Se	Pre-Se	MEL	Pre-MEL	SeNPs	Pre-SeNPs	MSeNPs (5 mg/kg)	MSeNPs (10 mg/kg)	Pre-MSeNPs (10 mg/kg)
Hb	g/dl	0.83 ± 0.16	1.3 ± 0.05 ^ns^	8.06 ± 0.06 **	9.3 ± 0.65 ***	10.7 ± 0.35 ****	7.4 ± 0.7 *	11.9 ± 0.1 ****	9.9 ± 0.95 ***	9.2 ± 0.15 ***	5.1 ± 0.45 ^ns^
RBCs	10^6^/µl	1.56 ± 0.29	5.16 ± 0.33 ^ns^	6 ± 0.28 ^ns^	6.4 ± 0.07 ^ns^	6.9 ± 0.28 ^ns^	5.07 ± 0.07 ^ns^	7.35 ± 0.35 *	6.6 ± 0.55 ^ns^	6.2 ± 0.23 ^ns^	7.2 ± 0.25 *
WBCs	10^3^/µl	90.7 ± 0.9	60.1 ± 1.15 ****	12.2 ± 0.61 ****	11.7 ± 0.35 ****	10.1 ± 0.1 ****	9.9 ± 0.95 ****	6.9 ± 0.05 ****	6.5 ± 0.5 ****	5.2 ± 0.2 ****	1.2 ± 0.2 ****
Hematocrit	%	14 ± 0.57	3.6 ± 0.3 ****	26.6 ± 0.6 ****	34.3 ± 1.16 ****	38.2 ± 0.61 ****	26.3 ± 0.65 ****	39.6 ± 0.87 ****	37.9 ± 1.15 ****	36.3 ± 0.3 ****	13.2 ± 0.61 ^ns^
Mean corpuscular volume	fL	54.7 ± 0.9	51.9 ± 1.56 ^ns^	53.7 ± 0.9 ^ns^	53.6 ± 0.87 ^ns^	54.7 ± 1.35 ^ns^	52 ± 1 ^ns^	53.9 ± 0.49 ^ns^	57.5 ± 0.5 ^ns^	58.3 ± 4.38 ^ns^	50.1 ± 0.1 ^ns^
Mean corpuscular hemoglobin	pg	17.5 ± 0.76	18.5 ± 0.28 ^ns^	14.4 ± 0.7 ^ns^	14.9 ± 0.49 ^ns^	15.3 ± 0.65 ^ns^	14.5 ± 0.5 ^ns^	16.1 ± 0.1 ^ns^	15 ± 1 ^ns^	14.7 ± 2.6 ^ns^	11.6 ± 0.83 *
Platelet count	10^3^/µl	45 ± 0.57	87 ± 1 ****	272 ± 6.1 ****	467 ± 1 ****	499 ± 2.08 ****	308 ± 1.52 ****	205 ± 4.04 ****	327 ± 3.51 ****	501 ± 0.57 ****	428 ± 1.5 ****
Neutrophils	%	83 ± 0.57	67 ± 1.52 ****	14.4 ± 0.4 ****	21.9 ± 0.49 ****	22.3 ± 0.3 ****	22.6 ± 0.83 ****	17.2 ± 0.61 ****	11.3 ± 0.33 ****	6.9 ± 0.95 ****	21.5 ± 0.56 ****
Lymphocytes	%	8.8 ± 0.7	21.4 ± 1.06 ****	71.7 ± 0.9 ****	61.7 ± 0.7 ****	58.6 ± 0.87 ****	62.9 ± 3.03 ****	77.1 ± 1.15 ****	75.3 ± 0.88 ****	90.4 ± 5.7 ****	74.8 ± 0.2 ****

EAC: Ehrlich Ascites Carcinoma; Se: treatment of selenium; pre-Se: pre-treatment of selenium; MEL: treatment of melatonin; pre-MEL: pre-treatment of melatonin; SeNPs: treatment of selenium nanoparticles; pre-SeNPs: pre-treatment of selenium nanoparticles; MSeNPs: melatonin-selenium nanoparticles; pre-MSeNPs: Pre-treatment of melatonin-selenium nanoparticles. Data are presented as mean ± SE. ns = non-significant, *=0.01, **=0.003, ***=0.0004, ****=0.0001.

### In vivo tumor growth inhibition and evaluation of MSeNPs’ antitumor activity

The highest cell viability was observed in the untreated EAC-bearing mice group, which was significantly higher than in all treated groups (*p* < 0.0001). Treatment with SeNPs reduced cell viability compared to sodium selenite. MSeNPs at a dosage of 10 mg/kg caused a further reduction in viable cells, showing significant improvements over sodium selenite (*p* < 0.0001), SeNPs (*p* < 0.0001), and MEL (*p* < 0.0001). Similarly, MSeNPs at a dose of 5 mg/kg resulted in fewer viable cells than sodium selenite (*p* < 0.0001), SeNPs (*p* < 0.0001), and MEL (*p* = 0.0004). There was no statistically significant variation between the 5 mg/kg and 10 mg/kg MSeNPs doses.

**Fig. 7:**
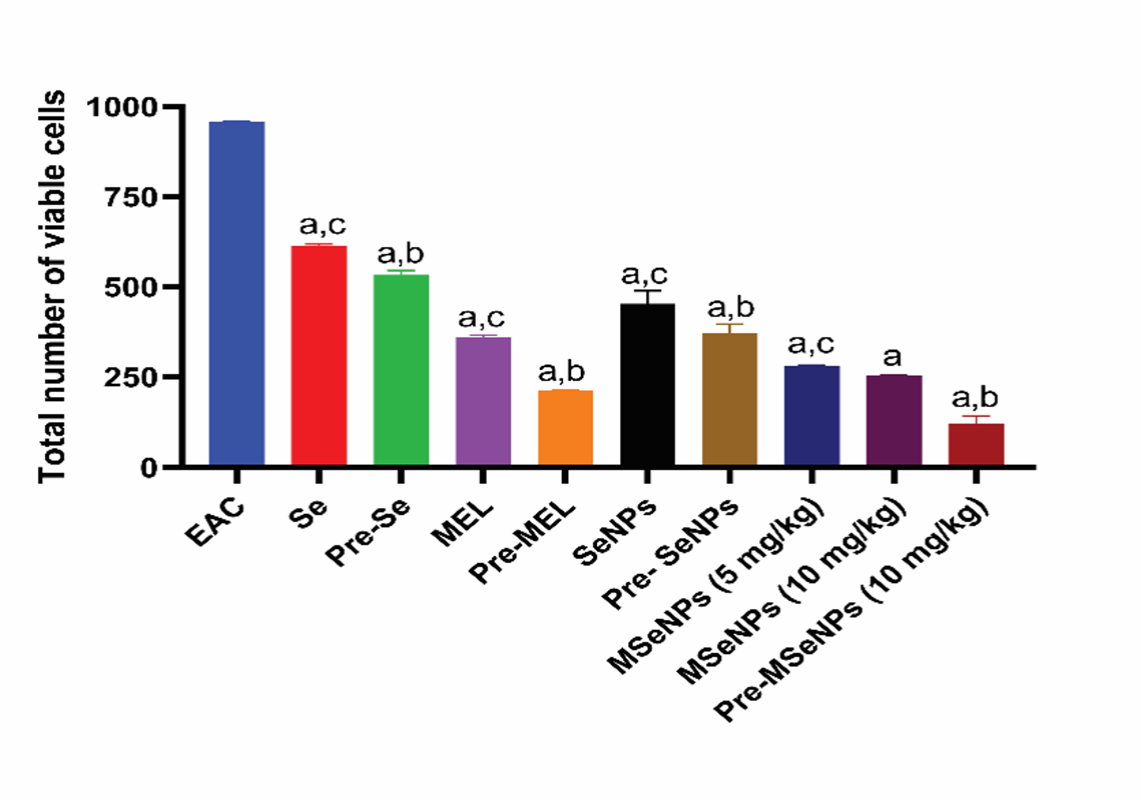
Number of viable cells in EAC-bearing mice in different groups. EAC: Ehrlich Ascites Carcinoma; Se: treatment of selenium; Pre-Se: Pre-treatment of selenium; MEL: treatment of melatonin; Pre-MEL: Pre-treatment of melatonin; SeNPs: treatment of selenium nanoparticles; pre-SeNPs: Pre-treatment of selenium nanoparticles; MSeNPs: melatonin-selenium nanoparticles; pre-MSeNPs: Pre-treatment of melatonin-selenium nanoparticles. Different letters indicate significant differences at *p* < 0.0001. a: all groups vs. EAC-bearing mice control group; b: pre-treatment groups vs. their corresponding treatment groups; c: treatment groups vs. MSeNPs at 10 mg/kg. Data are presented as mean ± SE.

In the pre-treatment groups, cell viability was lower than in the corresponding treatment groups. Pre-treatment with MSeNPs at 10 mg/kg resulted in the lowest viability, which was significantly lower than MSeNPs treatment at both 5 mg/kg and 10 mg/kg (*p* < 0.0001). Pre-treatment with sodium selenite was more effective than its treatment counterpart (*p* = 0.0002), while MEL pre-treatment was also more effective than MEL treatment (*p* < 0.0001). Similarly, pre-treatment with SeNPs showed greater efficacy than SeNP treatment (*p* = 0.0002). These results indicate that MSeNPs, particularly at 10 mg/kg as a pre-treatment, exert the most potent cytotoxic effect against EAC cells. These findings are presented in Fig. 7.

### Effect of MSeNPs on antioxidants in EAC-bearing mice in different groups

**Fig. 8:**
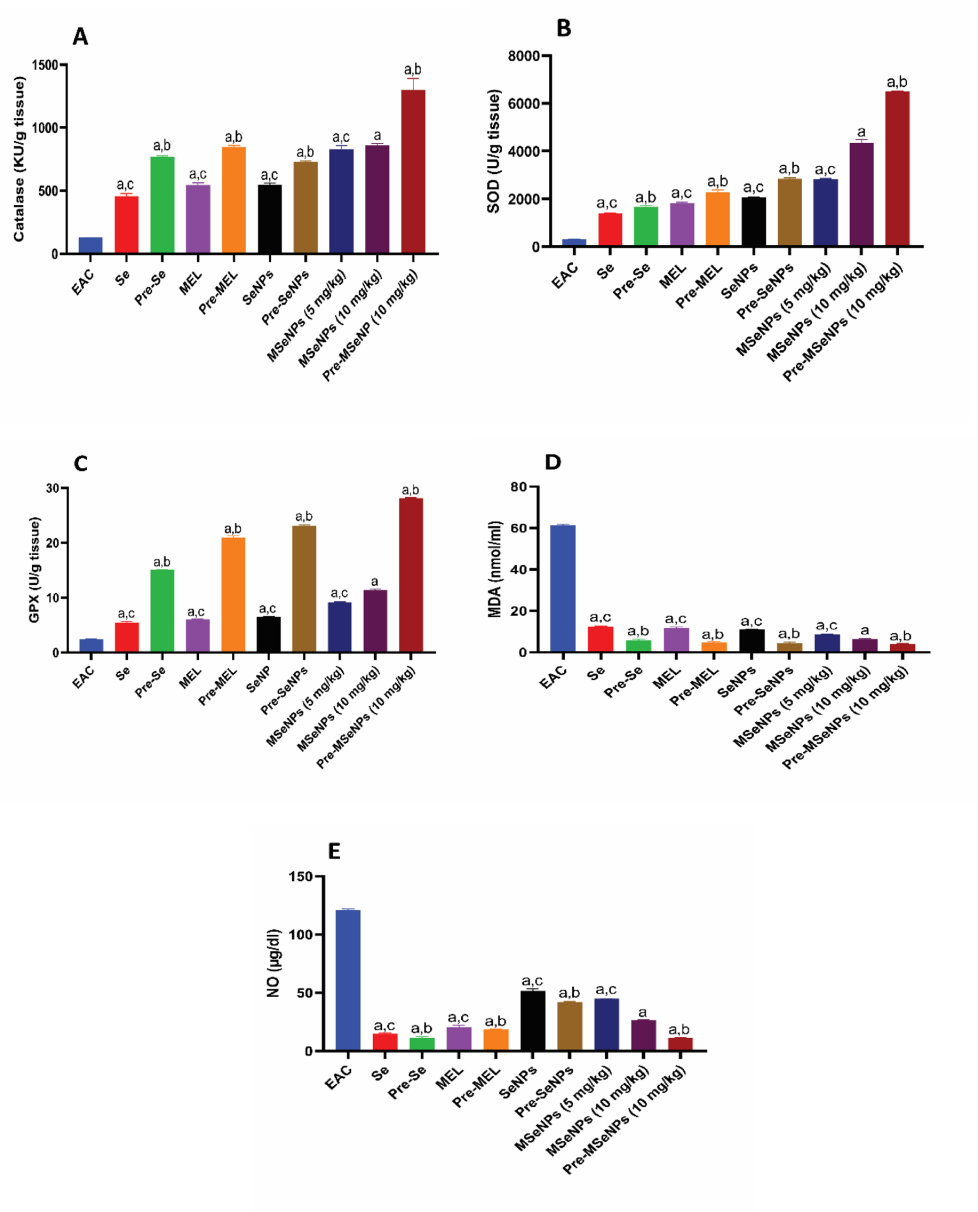
Effect of MSeNPs on antioxidants in EAC-bearing mice in different groups. (**A**) CAT (KU/g tissue), (**B**) SOD (U/g tissue), (**C**) GPX (U/g tissue), (**D**) Malondialdehyde (nmol/ml), (**E**) NO (µg/dl). Data are presented as mean ± SE.

The EAC-bearing control group exhibited the lowest CAT activity (*p* < 0.0001). Treatment with MSeNPs at both doses significantly increased CAT activity compared to sodium selenite, MEL, and SeNPs (*p* < 0.0001). No significant difference was observed between SeNPs and sodium selenite, nor between MSeNPs at 5 mg/kg and 10 mg/kg. Pre-treatment with MSeNPs at 10 mg/kg significantly enhanced CAT activity compared to pre-treatment with sodium selenite, MEL, and SeNPs (*p* < 0.0001). All pre-treatment groups exhibited significantly higher CAT activity than their corresponding treatment groups (*p* < 0.0001), except for the comparison between pre-treatment and treatment with SeNPs, where the difference was less pronounced (*p* = 0.01) (Fig. 8A). The EAC-bearing control group showed the lowest SOD activity (*p* < 0.0001). Treatment with SeNPs resulted in significantly higher SOD activity than sodium selenite alone (*p* < 0.0001). MSeNPs at 10 mg/kg significantly outperformed all other treatment groups (*p* < 0.0001), while MSeNPs at 5 mg/kg also demonstrated significant improvement over sodium selenite, MEL, and SeNPs (*p* < 0.0001). In the pre-treatment groups, MSeNPs at 10 mg/kg showed significantly higher SOD activity than all other pre-treatments (*p* < 0.0001). All pre-treatment groups exhibited significantly higher SOD activity than their corresponding treatment groups (*p* < 0.0001) (Fig. 8B).

GPX activity was lowest in the EAC-bearing control group (*p* < 0.0001). Treatment with SeNPs significantly increased GPX activity compared to sodium selenite alone (*p* = 0.002). MSeNPs at 10 mg/kg produced significantly higher GPX activity than all other treatment groups (*p* < 0.0001), while MSeNPs at 5 mg/kg also demonstrated significant improvement (*p* < 0.0001). In the pre-treatment groups, MSeNPs at 10 mg/kg exhibited significantly higher GPX activity than all other pre-treatments (*p* < 0.0001). All pre-treatment groups showed significantly higher GPX activity than their corresponding treatment groups (*p* < 0.0001) (Fig. 8C). All treatment and pre-treatment groups exhibited significantly lower MDA levels compared to the EAC-bearing control group (*p* < 0.0001). Within the treatment groups, SeNPs showed lower MDA levels than sodium selenite (*p* = 0.002). MSeNPs at 10 mg/kg produced significantly greater reductions in MDA compared to sodium selenite, MEL, SeNPs, and MSeNPs at 5 mg/kg (*p* < 0.0001), while MSeNPs at 5 mg/kg also demonstrated lower MDA levels than sodium selenite, MEL, and SeNPs (*p* < 0.0001). In the pre-treatment groups, MSeNPs at 10 mg/kg showed lower MDA levels than sodium selenite (*p* = 0.0007), with no significant difference compared to SeNPs and MEL. All pre-treatment groups exhibited significantly lower MDA levels than their corresponding treatment groups (*p* < 0.0001) (Fig. 8D).

All treatment and pre-treatment groups exhibited reduced NO levels compared to the EAC-bearing control group (*p* < 0.0001). Within the treatment groups, NO levels were lower in mice receiving sodium selenite, MEL, SeNPs, MSeNPs at 5 mg/kg, and MSeNPs at 10 mg/kg (*p* < 0.0001). In the pre-treatment groups, further reductions were observed, with MSeNPs at 10 mg/kg producing the most pronounced decrease compared to MEL and SeNPs (*p* < 0.0001), while showing no significant difference compared to sodium selenite. Comparisons between pre-treatment and corresponding treatment groups indicated significantly lower NO levels in pre-treated mice for sodium selenite (*p* = 0.0002), MEL (*p* = 0.01), and SeNPs (*p* < 0.0001). Notably, MSeNPs at 10 mg/kg as a pre-treatment resulted in significantly lower NO levels than both MSeNPs treatment groups (*p* < 0.0001), demonstrating superior efficacy in reducing NO levels (Fig. 8E).

### Effect of MSeNPs on IL-6 in EAC-bearing mice in different groups

**Fig. 9:**
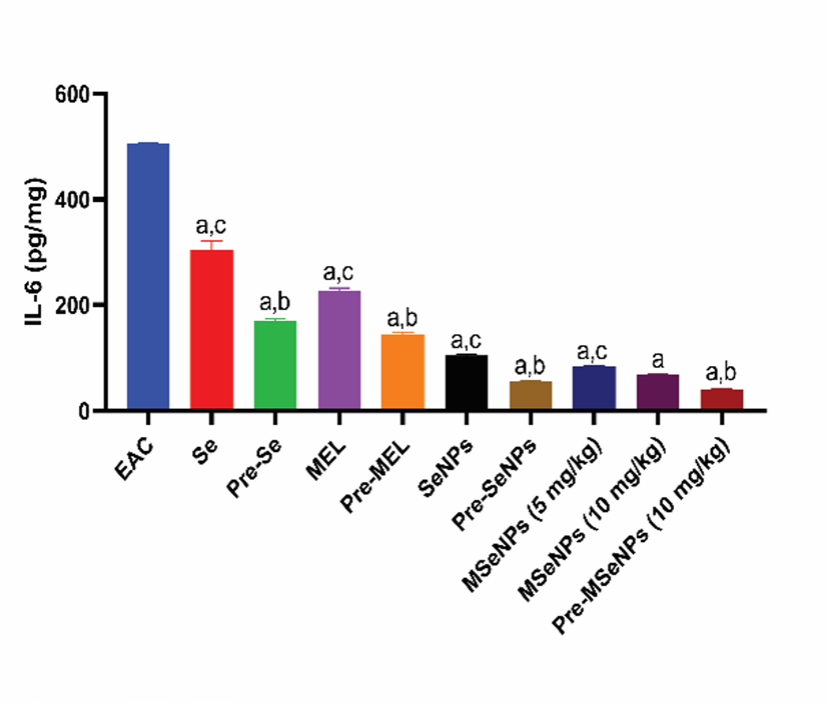
Effect of MSeNPs on IL-6 in EAC-bearing mice in different groups. Data are presented as mean ± SE.

Analysis of IL-6 levels in the ascitic fluid revealed that the EAC-bearing control group exhibited the highest IL-6 concentration (*p* < 0.0001). Treatment with SeNPs significantly reduced IL-6 levels compared to sodium selenite alone (*p* = 0.003). MSeNPs at 10 mg/kg markedly decreased IL-6 levels relative to sodium selenite, MEL, and SeNPs treatments (*p* < 0.0001), there was no significant variation between MSeNPs at 10 mg/kg and 5 mg/kg. MSeNPs at 5 mg/kg also significantly reduced IL-6 levels compared to sodium selenite and MEL (*p* < 0.0001) but did not differ significantly from SeNPs. In the pre-treatment groups, MSeNPs at 10 mg/kg significantly reduced IL-6 levels compared to sodium selenite and MEL (*p* < 0.0001), with no significant difference relative to SeNPs. All pre-treatment groups exhibited significantly lower IL-6 levels than their corresponding treatment groups: sodium selenite (*p* < 0.0001), MEL (*p* < 0.0001), SeNPs (*p* = 0.0001), and MSeNPs at 10 mg/kg versus MSeNPs at 5 mg/kg (*p* = 0.0006). No significant difference was observed between MSeNPs at 10 mg/kg in the treatment versus pre-treatment groups (Fig. 9).

### Analysis of the cell cycle in Ehrlich ascites tumor cells

Analysis of cell cycle distribution revealed that the EAC control group exhibited active proliferation, with high percentages of cells in the S and G2/M phases. Treatment with sodium selenite increased the proportion of cells in G0/G1 while reducing the S and G2/M phases. Pre-treatment with sodium selenite further elevated G0/G1 but unexpectedly increased the S-phase population, suggesting enhanced DNA replication potential. MEL treatment and pre-treatment both caused substantial G0/G1 arrest and strong suppression of S-phase, with pre-treatment producing even more pronounced effects. Similarly, SeNPs treatment increased G0/G1 and decreased S and G2/M phases, while pre-treatment with SeNPs intensified G0/G1 arrest and further suppressed the S-phase population.

**Fig. 10:**
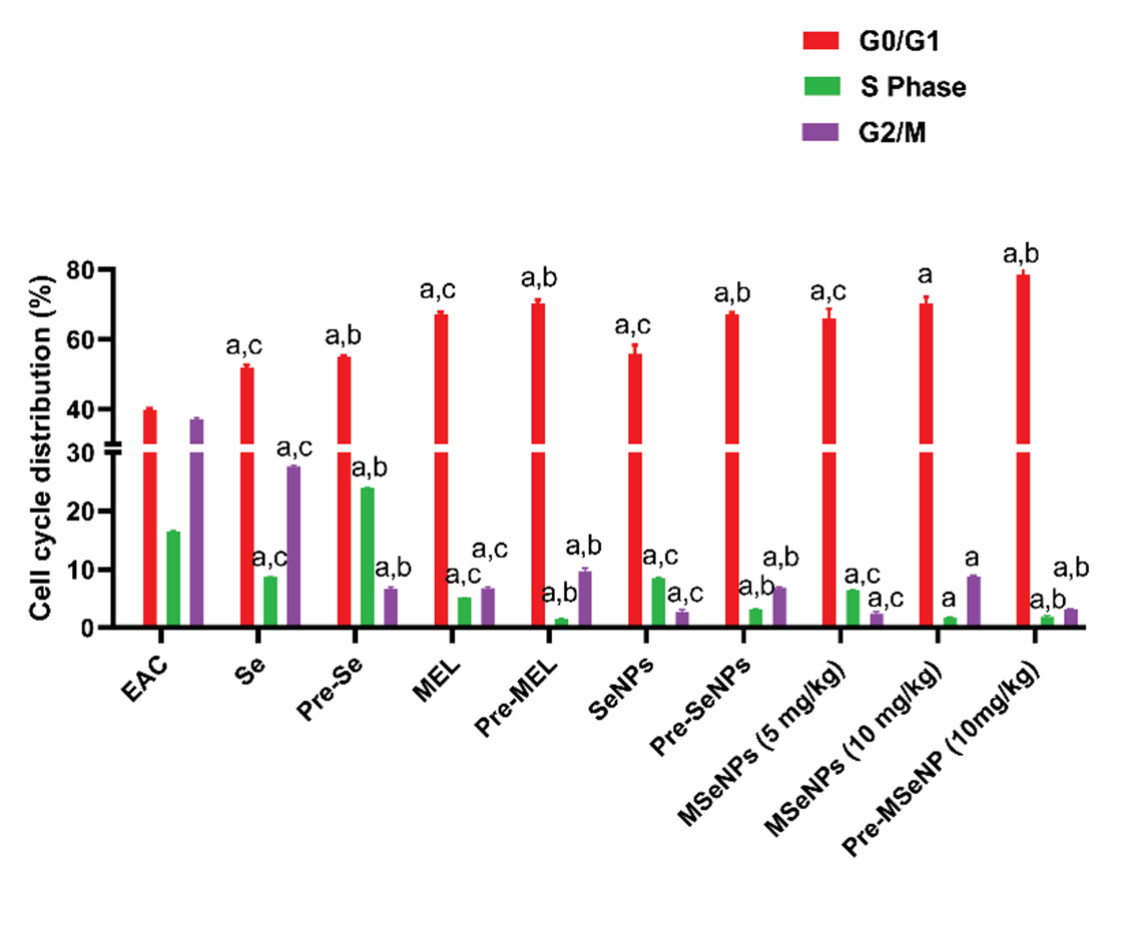
Cell cycle distribution of tumor cells in the different studied animal groups. EAC and the histograms of cell cycle. Different letters indicate significant differences at *p* < 0.0001. a: all groups vs. EAC-bearing mice control group; b: pre-treatment groups vs. their corresponding treatment groups; c: treatment groups vs. MSeNPs at 10 mg/kg. Data are presented as mean ± SE.

MSeNPs at 5 mg/kg increased the proportion of cells in G0/G1 and reduced both S and G2/M phases. MSeNPs at 10 mg/kg produced even stronger G0/G1 arrest, with near-complete suppression of the S-phase. Pre-treatment with MSeNPs at 10 mg/kg exhibited the most pronounced effects, showing the highest G0/G1 arrest (78.65%), the lowest S-phase percentage, and significantly reduced G2/M, indicating maximal suppression of proliferation. Overall, pre-treatment groups demonstrated stronger cell cycle arrest than the corresponding treatment groups, with pre-treatment using MSeNPs at 10 mg/kg achieving the greatest anti-proliferative effect (Fig. 10).

### Analysis of caspase-3 expression and apoptosis in Ehrlich ascites tumor cells

Analysis of caspase-3 expression revealed significant differences in apoptosis between the groups. Apoptotic activity was lowest in the EAC control group, with only 9.06% caspase-3-positive cells. Treatment with sodium selenite moderately increased caspase-3 positivity to 28.26% (*p* < 0.0001 vs. control), while pre-treatment with sodium selenite further raised this to 31.18% (*p* = 0.012 vs. Se treatment), indicating a modest enhancement of apoptosis. MEL treatment induced a stronger apoptotic response, with 52.56% caspase-3-positive cells (*p* < 0.0001 vs. control), and pre-treatment with MEL further increased apoptosis to 62.13% (*p* = 0.005 vs. MEL treatment).

**Fig. 11:**
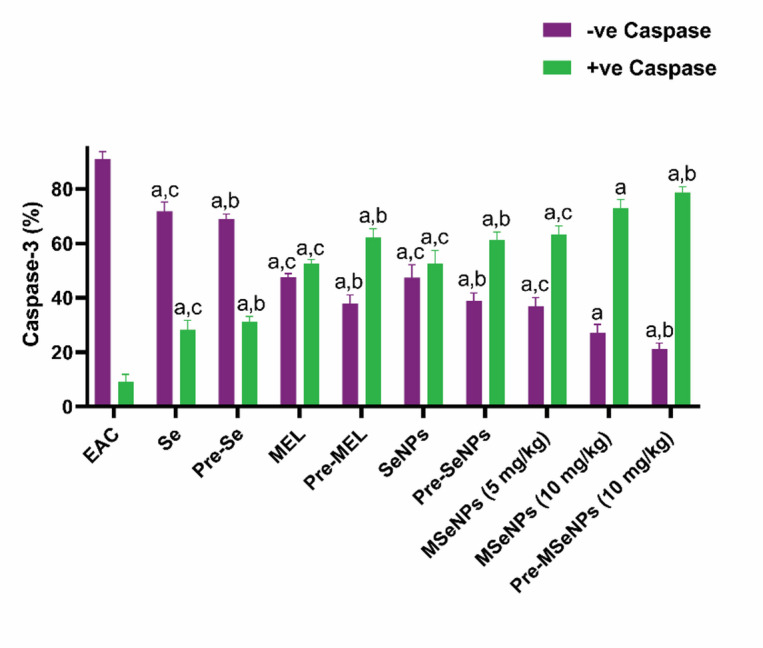
Expression of Caspase-3 in the tumor cells across the different studied animal groups. Caspase-Negative (− ve) Cells express viable (non-apoptotic) cells, Caspase-Positive (+ ve) Cells express apoptotic cells. Different letters indicate significant differences at *p* < 0.0001. a: all groups vs. EAC-bearing mice control group; b: pre-treatment groups vs. their corresponding treatment groups; c: treatment groups vs. MSeNPs at 10 mg/kg. Data are presented as mean ± SE.

SeNPs treatment induced 52.58% caspase-3 positivity (*p* < 0.0001 vs. control), while pre-treatment with SeNPs further increased it to 61.2% (*p* = 0.008 vs. SeNPs treatment). A more pronounced apoptotic effect was observed with MSeNPs: treatment with 5 mg/kg resulted in 63.16% caspase-3 positivity (*p* < 0.0001 vs. control), which increased to 68.93% at 10 mg/kg (*p* = 0.015 vs. MSeNPs at 5 mg/kg). Pre-treatment with MSeNPs at 10 mg/kg produced the highest caspase-3 positivity at 76.75% (*p* = 0.004 vs. MSeNPs 10 mg/kg treatment), indicating that this formulation and dosing regimen provides the most potent pro-apoptotic effect. Collectively, these results demonstrate that both treatment and pre-treatment with sodium selenite, MEL, and particularly MSeNPs significantly promote apoptosis in EAC-bearing mice, with pre-treatment using MSeNPs at 10 mg/kg exhibiting superior efficacy (Fig. 11).

### Expression of Ki-67 in Ehrlich ascites tumor cells

Analysis of Ki-67, a marker of cellular proliferation, revealed significant differences among the groups. Ki-67 expression was highest in the EAC control group (90.83%), indicating aggressive tumor proliferation. Treatment with sodium selenite reduced Ki-67 levels to 55.83% (*p* < 0.0001 vs. control), while pre-treatment with sodium selenite further suppressed proliferation to 43.33% (*p* = 0.0004 vs. Se treatment). MEL treatment decreased Ki-67 expression to 46.66% (*p* < 0.0001 vs. control), with pre-treatment producing even stronger inhibition at 33.33% (*p* = 0.0001 vs. MEL treatment).

**Fig. 12:**
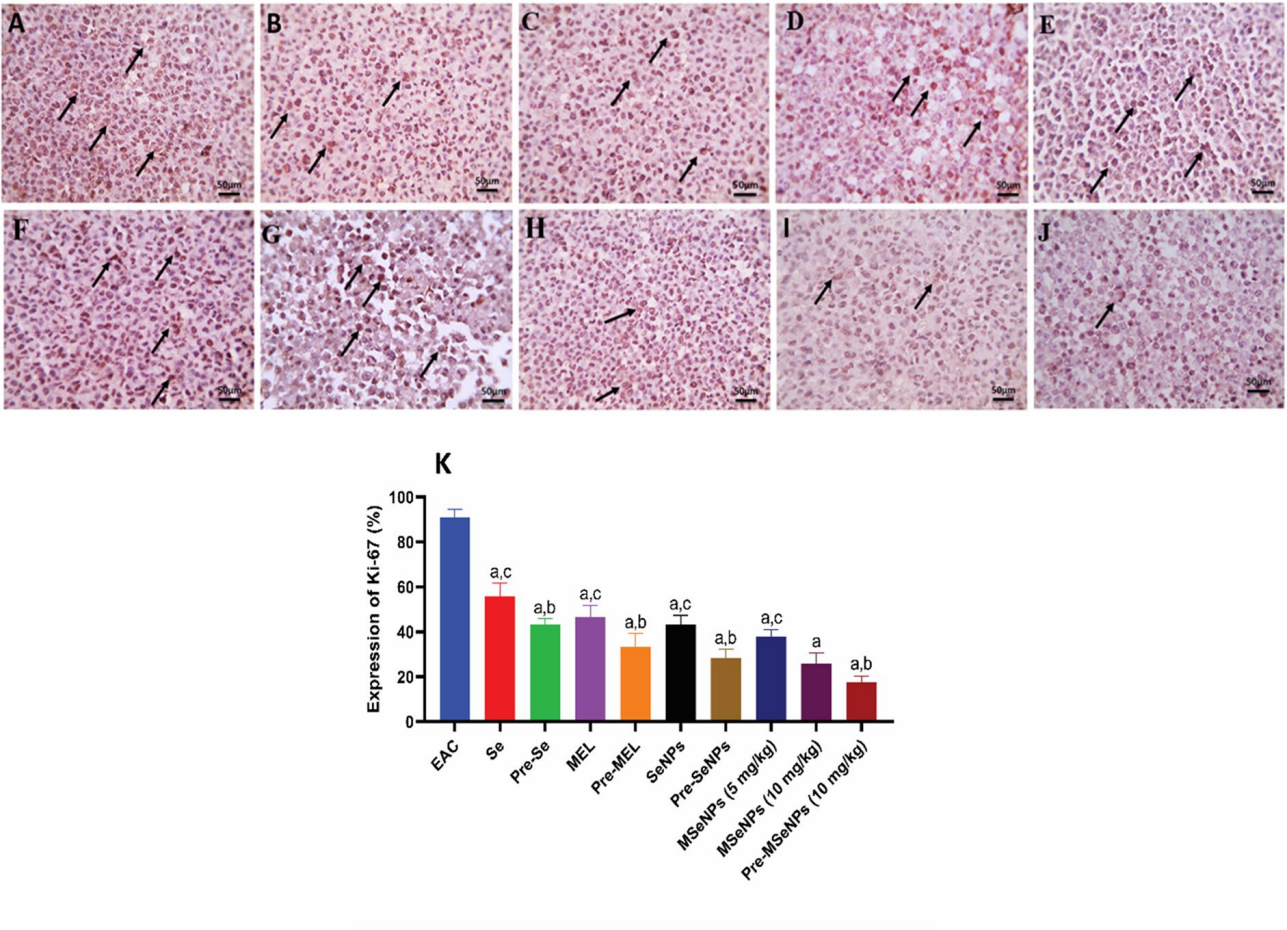
Microscopic images of immunostained sections of Ehrlich tumor cells against Ki-67 (**A**) EAC control, (**B**) treatment of Se, (**C**) pre-treatment of Se, (**D**) treatment of MEL, (**E**) pre-treatment of MEL, (**F**) treatment of SeNPs, (**G**) pre-treatment of SeNPs, (**H**) treatment of MSeNPs at 5 mg/kg, (**I**) treatment of MSeNPs at 10 mg/kg, (**J**) pre-treatment of MSeNPs at 10 mg/kg. The images show strong positive brown staining in tumor cells (black arrows) in the control group. Positive brown staining is reduced in all treatment groups, with the lowest staining observed in the MSeNPs at 10 mg/kg treatment group (black arrows). In pre-treatment groups, staining is further decreased, reaching the lowest level in the MSeNPs at 10 mg/kg pre-treatment group. (**K**) Quantitative measurement of Ki-67 in the Ehrlich Ascites tumor cells (magnification ×400, scale bar = 50 μm).

SeNPs exhibited a more pronounced anti-proliferative effect, with Ki-67 positivity of 43.33% in the treatment group (*p* < 0.0001 vs. control) and 28.33% in the pre-treatment group (*p* < 0.0001 vs. SeNPs treatment). The MSeNPs treatment groups demonstrated the most potent inhibition of proliferation: MSeNPs at 5 mg/kg reduced Ki-67 expression to 37.83% (*p* < 0.0001 vs. sodium selenite; *p* = 0.03 vs. MEL), while MSeNPs at 10 mg/kg further decreased Ki-67 levels to 25.83% (*p* < 0.0001 vs. all other treatment groups; *p* = 0.0007 vs. MSeNPs 5 mg/kg). The greatest suppression of proliferation was observed in the pre-treatment group with MSeNPs at 10 mg/kg, which showed only 17.5% Ki-67 positivity. This value was significantly lower than all other pre-treatment groups, including sodium selenite (*p* < 0.0001), MEL (*p* < 0.0001), and SeNPs (*p* = 0.003), and even lower than the MSeNPs 5 mg/kg treatment group (*p* < 0.0001) (Fig. 12). These findings were corroborated by immunohistochemical analysis, in which the intensity and extent of brown nuclear staining, indicative of Ki-67 expression, were highest in the EAC control group and progressively decreased across the treatment and pre-treatment groups. The most pronounced reduction was observed in the pre-treatment group receiving MSeNPs at 10 mg/kg, confirming that this formulation and dosing regimen exerts the strongest anti-proliferative effect.

### Histopathological analysis and pathological changes in tumor cells and kidney tissues

**Fig. 13:**
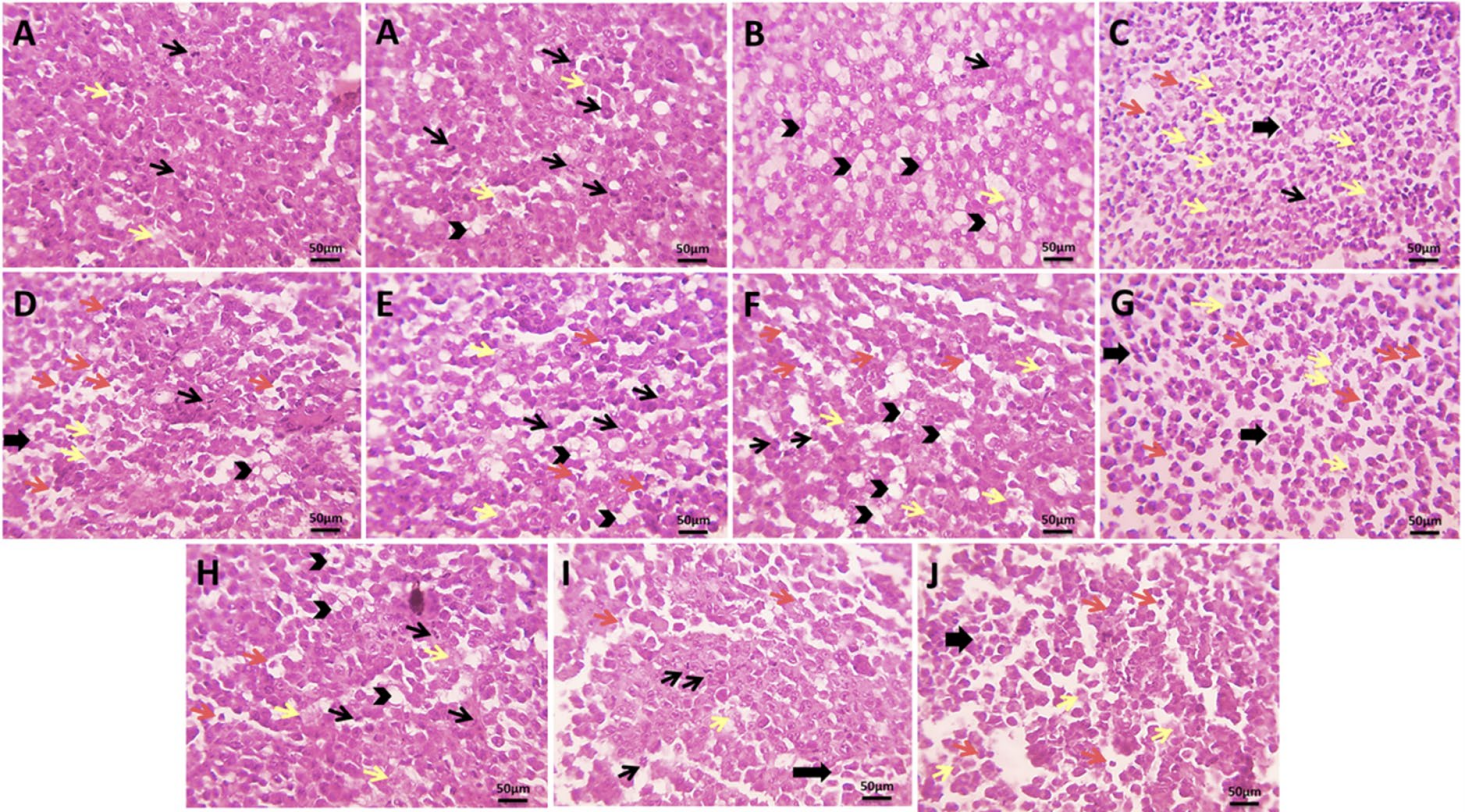
Microscopic pictures of H&E-stained of EAC cells. (**A**) Untreated EAC control group, (**B**) treatment of Se, (**C**) pre-treatment of Se, (**D**) treatment of MEL, (**E**) pre-treatment of MEL, (**F**) treatment of SeNPs, (**G**) pre-treatment of SeNPs, (**H**) treatment of MSeNPs at 5 mg/kg, (**I**) treatment of MSeNPs at 10 mg/kg, (**J**) pre-treatment of MSeNPs at 10 mg/kg. Arrowheads indicate vacuolated tumor cells; thin black arrows, mitotic figures; yellow arrows, necrotic ghost cells; red arrows, apoptotic cells (magnification ×400, scale bar = 50 μm).

Histopathological examination of EAC tumor sections revealed marked differences in tumor cell morphology and growth patterns among the experimental groups. The EAC control group exhibited aggressive tumor proliferation, characterized by numerous mitotic figures and minimal necrosis. Treatment with sodium selenite or MEL produced modest reductions in tumor growth, while pre-treatment with either agent resulted in more pronounced effects, including increased necrosis and apoptosis. SeNPs treatment induced moderate tumor suppression, which was further enhanced in the pre-treatment group. MSeNPs treatment at 10 mg/kg elicited stronger anti-tumor effects, and pre-treatment with MSeNPs at 10 mg/kg produced the most significant tumor regression, evidenced by an absence of mitotic figures, extensive necrosis, and widespread apoptotic cells (Fig. 13).

Pathological assessment showed minimal damage in the EAC control group. Slight to moderate changes were observed in the sodium selenite, MEL, and SeNPs groups. Pre-treatment groups exhibited stronger effects, with pre-treatment using MSeNPs at 10 mg/kg causing the most severe tumor damage, characterized by extensive necrosis, widespread apoptosis, and complete absence of mitotic activity, indicating the strongest anti-tumor effect. MEL and SeNPs demonstrated greater efficacy compared to their conventional forms, likely due to improved bioavailability and synergistic effects. Notably, pre-treatment with MSeNPs at 10 mg/kg produced the most pronounced tumor cell necrosis and apoptosis, along with complete inhibition of mitotic activity (Table 2).

**Table 2:** Pathological changes in tumor cells.

Pathological changes indicating retarded growth	A	B	C	D	E	F	G	H	I	J
Necrosis	+	+	++	+	++	++	++	++	++	+++
Apoptosis	+	−	++	++	++	++	++	++	++	+++
Vacuolation	−	+++	++	−	−	++	−	+	+	−
Shrinkage	−	−	−	++	+++	−	++	−	+	++
Scoring of Mitosis	+++	+	++	++	+	++	−	++	+	−

A; untreated EAC control group, B; treatment of Se, C; pre-treatment of Se, D; treatment of MEL, E; pre-treatment of MEL, F; treatment of SeNPs, G; pre-treatment of SeNPs, H; treatment of MSeNPs at 5 mg/kg, I; treatment of MSeNPs at 10 mg/kg, J; pre-treatment of MSeNPs at 10 mg/kg. Histopathological changes were graded using a semi-quantitative scale as follows: absent (−), mild (+), moderate (++), and severe (+++).

**Fig. 14:**
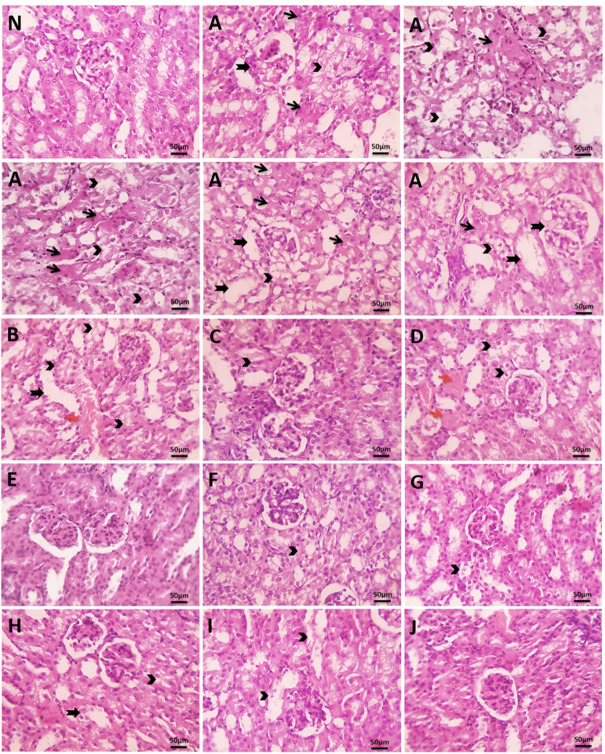
Microscopic pictures of H&E-stained kidney sections. N; negative control, (**A**) the untreated EAC bearing mice control group, (**B**) treatment of Se, (**C**) pre-treatment of Se, (**D**) treatment of MEL, (**E**) pre-treatment of MEL, (**F**) treatment of SeNPs, (**G**) pre-treatment of SeNPs, (**H**) treatment of MSeNPs at 5 mg/kg, (**I**) treatment of MSeNPs at 10 mg/kg, (**J**) pre-treatment of MSeNPs at 10 mg/kg. Thick black arrows indicate tubular dilation; thin black arrows, tubular epithelial degeneration; red arrows, congested intertubular capillaries; black arrowheads, coagulative necrosis (magnification ×400, scale bar = 50 μm).

Histopathological analysis of kidney tissue revealed severe damage in the untreated EAC control group, including extensive tumor cell aggregation, degenerated glomeruli, tubular dilation, and marked necrosis. In contrast, the negative control group showed normal kidney architecture with no observable pathological changes. Treatment with sodium selenite, MEL, SeNPs, and MSeNPs generally ameliorated these pathological changes, while pre-treatment groups exhibited milder capsular aggregation, reduced tubular dilation, and less hydropic degeneration. Among all groups, pre-treatment with MSeNPs at 10 mg/kg resulted in the least kidney damage, indicating a strong protective effect against EAC-induced renal injury (Fig. 14). The untreated EAC mice exhibited severe kidney damage, whereas treatment with sodium selenite, MEL, and SeNPs resulted in moderate histopathological changes. Pre-treatment groups, particularly those receiving MSeNPs at 10 mg/kg, showed the least kidney pathology, indicating strong protective effects. Overall, pre-treatment was more effective than direct treatment, and nanoparticle formulations provided superior renal protection compared to Se or MEL alone, as summarized in Table 3.

**Table 3:** Pathological changes in Kidney.

Pathological changes in kidneys	*N*	A	B	C	D	E	F	G	H	I	J
Vascular congestion/dilation	−	+	+	−	+	−	−	−	−	−	−
Capsular infiltration of EAC cells	−	+++	+	+	+	+	++	-	++	++	+
Tubular dilation	−	++	++	−	+	++	+	+	+	+	−
Tubular degeneration	−	+++	++	−	++	+	++	+	+	+	−
Tubular necrosis	−	++	−	−	−	−	−	+	−	−	−
Glomerular damage	−	++	−	−	−	−	−	−	−	−	−

N; negative control, A; untreated EAC control group, B; treatment of Se, C; pre-treatment of Se, D; treatment of MEL, E; pre-treatment of MEL, F; treatment of SeNPs, G; pre-treatment of SeNPs, H; treatment of MSeNPs at 5 mg/kg, I; treatment of MSeNPs at 10 mg/kg, J; pre-treatment of MSeNPs at 10 mg/kg. Histopathological changes were graded using a semi-quantitative scale as follows: absent (−), mild (+), moderate (++), and severe (+++).

## Discussion

Combining multiple drugs can simultaneously target different aspects of a disease, often enhancing therapeutic outcomes. In cancer therapy, the administration of anticancer agents with distinct mechanisms of action may improve overall efficacy compared to single-agent treatments. In this study, we evaluated the synergistic effects of MEL and SeNPs in an EAC-bearing mouse model. Our results demonstrated that the combined administration of MEL and SeNPs produced significantly better outcomes than either agent alone, indicating a promising synergistic effect. These results suggest that this combination may be a more successful approach to treating cancer. Combination therapy represents a powerful approach in oncology, since it not only improves therapeutic efficacy but also aids in overcoming medication resistance^33^. Furthermore, the use of SeNPs in combination with other therapeutic agents shows considerable promise for the development of next-generation anticancer treatments^34^.

Ascitic fluid serves as a direct nutritional source for tumor cells, and as these cells proliferate rapidly, the volume of ascitic fluid increases proportionally. In this study, intraperitoneal injection of EAC cells in mice led to the development of ascitic tumors, with significant accumulation of ascitic fluid observed within three weeks. Consistent with previous research, untreated tumor-bearing mice also exhibited a marked increase in ascitic fluid volume over time^25^. The present study demonstrated that MSeNPs exert significant antitumor effects, markedly reducing tumor volume. The immune system plays a role in promoting MEL production, while MEL, in turn, exhibits immunomodulatory effects^35^. MEL also demonstrates anti-inflammatory properties by decreasing the levels of tumor necrosis factor-alpha, interleukin-2, and interferon-gamma, while increasing the concentrations of interleukin-4, interleukin-10, and interleukin-27. MEL serves multiple physiological roles, notably its ability to neutralize ROS, reflecting its strong antioxidant capacity. In addition, it contributes to immune regulation, helps manage inflammatory conditions, and exhibits antiviral activity^36^.

MEL has been shown to inhibit tumor growth and the proliferation of cancer cells by inducing apoptosis, thereby reducing the likelihood of malignant transformation of healthy cells. Additionally, it supports cellular renewal by facilitating the removal of tumor cells and promoting their replacement with healthy cells through apoptotic pathways^37^. MEL promotes the upregulation of caspase-3 and -9 activity, thereby inducing apoptosis. Extensive research has demonstrated the anticancer potential of MEL in both leukemia and solid tumors, with particularly notable effectiveness against lymphoproliferative malignancies^38^. El Missiri et al. explored the impact of MEL on EAC cells and found that MEL not only induced the death of EAC tumor cells but also significantly extended the survival of treated experimental animals^39^. Cancer cells exhibit altered redox homeostasis, characterized by increased ROS production and impaired antioxidant defense mechanisms. MEL acts as potent antioxidant in normal cells by scavenging ROS, whereas in cancer cells it can disrupt redox balance further, contributing to oxidative stress-mediated apoptosis and exerting anticancer effects^40^.

One of the primary objectives of nanomedicine is to address the challenges commonly associated with traditional drug formulations, particularly by enhancing their safety profiles. In this context, numerous studies have highlighted the promising medical applications of SeNPs, which offer a range of therapeutic benefits, including antioxidant, anti-inflammatory, anti-diabetic, and anticancer activities^41^. It is well established that the pharmacological properties and toxicity of Se-based compounds are strongly influenced by their concentration, redox state, and chemical form^42^. Various anticancer mechanisms have been attributed to SeNPs, including the activation of apoptotic pathways^43^, generation of ROS^44^, induction of cell cycle arrest^45^, disruption of cellular homeostasis^46^, and mitochondrial dysfunction accompanied by DNA fragmentation^47^. These findings help explain our results and highlight the synergistic effect of MEL and SeNPs in treating cancer cells in the Ehrlich ascites carcinoma model, leading to the excellent outcomes presented above.

In the current study, the EAC group exhibited significant hematological abnormalities, including markedly reduced Hb, RBC counts, and platelet levels, along with substantially elevated WBC counts and an imbalanced immune cell profile characterized by increased neutrophils and decreased lymphocytes, indicative of tumor progression. Among the treatments, MSeNPs demonstrated the most favorable effects, effectively restoring Hb, RBCs, and platelet levels and achieving the highest lymphocyte percentage, suggesting strong hematological recovery. Notably, pre-treatment strategies further improved outcomes across all groups. These effects are attributed to the protective action of SeNPs and MEL on the hematopoietic system through their chemoprotective properties^48^.

A growing body of evidence supports the wide-ranging anticancer properties of MEL, including its ability to suppress cancer cell viability, proliferation, progression, metastasis, and even prevent cancer initiation. Numerous studies have highlighted the regulatory effects of MEL in cancer management, demonstrating its influence across multiple stages of the disease, including initiation, progression, and metastasis^49^. Interestingly, although the antitumor effects of Se have been well documented, they are often observed only at doses approaching toxicity. In contrast, SeNPs exhibit strong anticancer activity with reduced toxicity. Their free radical scavenging ability is size-dependent, with smaller nanoparticles showing greater efficacy in combating free radicals and shielding DNA from oxidative damage. Furthermore, when compared to other compounds containing Se, SeNPs have higher levels of bioavailability and biological activity^46^. These properties explain the observed cell viability results in this study: EAC-bearing mice showed the highest number of viable cells, whereas both treatment and pre-treatment groups with MSeNPs exhibited the lowest viable cell counts, outperforming MEL, Se, or SeNPs administered alone.

Oxidative stress is reported to be a key factor in the initiation and progression of cancer, as it promotes tumor development and enhances cellular proliferation. Consequently, the use of antioxidant agents represents a promising strategy for cancer prevention^50^. MSeNPs exhibit excellent antioxidant activity by modulating ROS. In our study, EAC-bearing mice demonstrated a significant reduction in antioxidant levels, including CAT, SOD, and GPx, consistent with previous reports^51^. Both treatment and pre-treatment groups with MSeNPs demonstrated upregulation of enzymatic antioxidant defense systems (CAT, SOD, and GPx), along with downregulation of oxidative stress biomarkers such as MDA and NO, in agreement with earlier studies^52^. Moreover, the nano-combination approach displayed more pronounced antioxidant properties^20^. Nanocarrier-based systems have been shown to enhance the stability and solubility of bioactive compounds, thereby improving their bioavailability^53^.

IL-6 is a multifunctional cytokine involved in regulating a wide range of homeostatic and pathological processes. Pathologically, tumor stromal cells, infiltrating immune cells, or the cancer cells themselves can produceIL-6^54^. It is abundantly present in the tumor microenvironment of various cancers, such as melanoma, breast cancer, ovarian cancer, non-small-cell lung cancer, pancreatic cancer, and head and neck squamous cell carcinoma. In this study, EAC-bearing mice exhibited a markedly elevated level of IL-6, whereas both treatment and pre-treatment groups with MSeNPs showed the lowest IL-6 levels among all groups. These results align with earlier research showing the antitumor effects of MEL^55^, and SeNPs^56^.

Targeting cell cycle progression in cancer cells represents a promising strategy for controlling tumor growth^57^. In our study, analysis of cell cycle distribution revealed that pre-treatment groups consistently exhibited stronger anti-proliferative effects compared to treatment groups. Notably, pre-treatment with MSeNPs at 10 mg/kg induced the most pronounced G0/G1 arrest, accompanied by near-complete suppression of the S-phase and a marked reduction in the G2/M phase, indicating maximal inhibition of cell cycle progression. This enhanced efficacy may be mechanistically attributed to redox-dependent differential sensitivity between cancerous and normal cells. Cancer cells typically exhibit elevated baseline levels of ROS along with weakened antioxidant capacity. Exposure to MSeNPs can further increase ROS production, pushing oxidative stress beyond the tolerable threshold and leading to cell cycle arrest and potentially the activation of apoptotic pathways. In contrast, normal cells possess more efficient antioxidant defense systems, allowing them to maintain redox homeostasis under similar conditions^46^. Collectively, these findings highlight a selective redox-mediated mechanism underlying the anti-proliferative action of MSeNPs. Consistent with these observations, the superior efficacy of the pre-treatment strategy and the potent effect of MSeNPs in enhancing cell cycle arrest and suppressing tumor cell proliferation. These results are consistent with previous studies reporting the antitumor and antiproliferative effects of MEL^37^, and SeNPs^46^.

Various enzymes have been explored as potential therapeutic targets for managing human diseases, with caspase-3 being particularly noteworthy. Caspase-3, encoded by the *CASP3* gene^58^, is a key mediator of apoptosis, activated in cells *via* both the extrinsic (death receptor) and intrinsic (mitochondrial) pathways. Beyond its role in apoptosis, caspase-3 also contributes to non-apoptotic processes, including tissue differentiation, regeneration, and neural development^59^. Flow cytometry analysis of caspase-3 expression revealed that all treatment and pre-treatment groups significantly enhanced apoptosis compared to the EAC control group. Notably, MEL, SeNPs, and especially MSeNPs treatments induced strong pro-apoptotic responses. Among these, MSeNPs at a dose of 10 mg/kg, particularly when used as a pre-treatment, demonstrated the highest caspase-3 positivity, indicating a superior ability to induce apoptosis compared to MEL or SeNPs alone. These findings highlight the therapeutic potential of MSeNPs, with dose-dependent and pre-treatment benefits, in enhancing apoptotic pathways in EAC-bearing mice. Our findings align with those of Wang and colleagues, who showed that MEL causes breast cancer cells to undergo apoptosis by stimulating the activities and cleavage of caspase-3 and caspase-9^60^, and with de Godoy, who demonstrated similar effects in breast cancer cell lines harboring *PIK3CA* gene mutations^61^. Furthermore, these findings align with previous research using SeNPs in hepatocellular carcinoma, which reported increased expression of several pro-apoptotic genes, including *CASP3* and *CASP4*^62^.

Ki-67 is a well-established proliferation marker commonly used in pathology to assess cellular proliferation rates^63^. Immunohistochemical analysis of our formulation demonstrated a more pronounced antitumor effect against EAC compared to MEL or SeNPs alone, as evidenced by markedly reduced Ki-67 expression. All treatment and pre-treatment groups significantly decreased Ki-67 levels compared to the EAC control, which exhibited the highest proliferation rate. Sodium selenite and MEL treatments moderately reduced Ki-67 expression, with greater suppression observed in their respective pre-treatment groups. SeNPs further enhanced this anti-proliferative effect. Most notably, MSeNPs treatments produced the strongest reduction in Ki-67 expression, particularly at 10 mg/kg with pre-treatment, which showed the greatest inhibition of tumor cell proliferation. Immunohistochemical staining corroborated these findings, revealing progressively diminished Ki-67 positivity and staining intensity across the groups, confirming the superior efficacy of MSeNPs in inhibiting EAC tumor proliferation. These results are consistent with previous studies demonstrating the apoptotic and anti-proliferative effects of SeNPs^64^, and MEL^65^.

Histopathological evaluation of EAC tumor tissues revealed that MSeNPs, particularly at 10 mg/kg with pre-treatment, produced the most pronounced anti-tumor effects. The control group exhibited aggressive tumor proliferation with numerous mitotic figures and minimal necrosis, whereas treatment with sodium selenite, MEL, and SeNPs resulted in moderate tumor suppression. Pre-treatment with these agents induced more pronounced tumor regression, with MSeNPs pre-treatment causing extensive necrosis, increased apoptosis, and complete absence of mitotic activity. These effects were markedly stronger than those observed with the conventional formulations, suggesting enhanced bioavailability and synergistic activity in the nanoparticle-based formulation.

Histopathological analysis of kidney tissues showed severe damage in the EAC control group. All treatments reduced renal pathology to varying degrees, with pre-treatment providing superior protection. Notably, the MSeNPs 10 mg/kg pre-treatment group exhibited minimal renal damage, indicating both strong anti-tumor efficacy and protection against EAC-induced kidney injury. These findings highlight the advantages of nanoparticle-based formulations and the importance of pre-treatment in enhancing therapeutic outcomes while reducing systemic toxicity. Consistent with previous studies, MEL has protective and therapeutic effects against renal diseases^66^, and SeNPs have been shown to prevent renal damage^67^. Despite these promising findings, this study is limited by the use of a single animal model and lack of long-term toxicity evaluation, which may limit the generalizability of the results. Future research is necessary to evaluate the therapeutic potential and long-term safety profile of MSeNPs by evaluating their efficacy and safety in a variety of cancer models and in clinical settings.

## Conclusion

This study provides evidence supporting the potential use of safe and stable MSeNPs as an antitumor agent in both the pre-treatment and treatment of EAC-bearing mice. MSeNPs were observed to suppress tumor growth by enhancing antioxidant defenses (CAT, SOD, and GPx) and reducing oxidative stress markers (MDA and NO). MSeNPs also significantly lowered IL-6 levels, contributing to the modulation of EAC-induced inflammation. Additionally, they promoted apoptosis *via* caspase-3 activation and reduced cellular proliferation through Ki-67 downregulation, accompanied by G0/G1 cell cycle arrest and a reduction in the S-phase population. Collectively, these results indicate that the combination of MEL and SeNPs may provide a more effective therapeutic approach than MEL, Se, or SeNPs alone.

## Supplementary Information

Below is the link to the electronic supplementary material.

Supplementary Material 1

## Data Availability

The datasets used and/or analyzed during the current study are available from the corresponding author on reasonable request.

## Abbreviations

EAC: Ehrlich ascites carcinoma
MEL: Melatonin
Se: Selenium
ROS: Reactive oxygen species
SeNPs: Selenium nanoparticles
NOAELs: No observed adverse effect levels
LOAELs: Lowest observed adverse effect levels
MSeNPs: Melatonin-selenium nanoparticles
SEM: Scanning electron microscope
TEM: Transmission electron microscope
EDX: Energy dispersive X-ray
Hb: Hemoglobin
RBCs: Red blood cells
WBCs: White blood cells
CAT: Catalase
SOD: Superoxide dismutase
GPX: Glutathione peroxidase
MDA: Malondialdehyde
NO: Nitric oxide
IL-6: Interleukin-6
H&E: Hematoxylin and eosin
SE: Standard error
