# Supplementary material for: Melatonin–selenium nanoformulation: a promising therapeutic strategy against Ehrlich ascites carcinoma
Source: Sci Rep. 2026 May 26;16:16264. doi: 10.1038/s41598-026-53359-w (PMC13212612; doi:10.1038/s41598-026-53359-w)
Supplement: Supplementary file 1 — Supplementary Material 1 [file 41598_2026_53359_MOESM1_ESM.docx]

**Supplementary Information**

Melatonin - Selenium Nanoformulation: A Promising Therapeutic Strategy Against Ehrlich Ascites Carcinoma

**Hanaa M. Morad^1^,** **A.F. Abdel-Aziz^1^, Mai M. Madkour^1*^**

*¹* Department of Chemistry, Faculty of Science, Mansoura University, Mansoura 35516, Egypt

*Authors of correspondence:

A.F. Abdel-Aziz ([afaziz2012@hotmail.com](mailto:afaziz2012@hotmail.com))

Mai M. Madkour ([maimadkour211@mans.edu.eg](mailto:maimadkour211@mans.edu.eg))

**Combination Index (CI) Analysis**

The combination index (CI) was calculated using the Chou-Talalay method to assess drug interactions at different doses.

- Doses: 5 mg/kg and 10 mg/kg
- Melatonin (MEL): 95%
- Selenium (Se): 1%

For 5 mg/kg:

- Melatonin (MEL) = 5 × 0.95 = 4.75 mg/kg
- Selenium (Se) = 5 × 0.01 = 0.05 mg/kg
- CI = (4.75 / 10) + (0.05 / 10) = 0.48

For 10 mg/kg:

- Melatonin (MEL) = 10 × 0.95 = 9.5 mg/kg
- Selenium (Se) = 10 × 0.01 = 0.1 mg/kg
- CI = (9.5 / 10) + (0.1 / 10) = 0.96

These results indicate that at 5 mg/kg, the combination demonstrates a synergistic effect (CI < 1), whereas at 10 mg/kg, the interaction is nearly additive with slight synergy (CI = 1).

**Dose Calculation on a Mass Basis**

Doses were calculated based on the mass of MSeNPs relative to animal body weight. The mean body weight of the mice was 25 g (0.025 kg).

- MSeNPs (5 mg/kg): 0.125 mg per mouse
- MSeNPs (10 mg/kg): 0.25 mg per mouse

**Vehicle Composition**

MSeNPs were formulated in a vehicle consisting of 8% dimethyl sulfoxide (DMSO) and 92% saline. This composition ensured drug stability and maintained bioavailability throughout the study.

**Sterility and Endotoxin Testing**

All formulations were prepared under aseptic conditions using sterile glassware and consumables. Sterility and endotoxin contamination were assessed according to standard protocols, confirming that the preparations were free from microbial contamination and that endotoxin levels were within acceptable limits. Endotoxin-free water and reagents were used throughout to ensure safety and reliability.

**Table S(1)** Effect of MSeNPs on tumor volume in EAC-bearing mice in different groups

| **Group**  **Parameter** | **EAC** | **Se** | **Pre-Se** | **MEL** | **Pre-MEL** | **SeNPs** | **Pre-SeNPs** | **MSeNPs**  **(5 mg/kg)** | **MSeNPs**  **(10 mg/kg)** | **Pre-MSeNPs**  **(10 mg/kg)** |
| --- | --- | --- | --- | --- | --- | --- | --- | --- | --- | --- |
| **Tumor volume**  **(cm^3^)** | 15.17±0.12 | 14.17±0.29^a,c^ | 12.31±0.29^a,b^ | 10.94±0.16^a,c^ | 9.43±0.34 ^a,b^ | 8.79±0.19^a,c^ | 7.85±0.44^a,b^ | 3.73±0.22^a,c^ | 2.73±0.18^a^ | 2.41±0.14^a,b^ |

Different letters indicate significant differences at p < 0.0001. a: all groups vs EAC bearing mice control group; b: pre-treatment groups vs its related treatment ones; c: treatment groups vs MSeNPs at dose 10 mg/kg.

**Table S(2)** Number of viable cells in EAC-bearing mice in different groups

| **Group**  **Parameter** | **EAC** | **Se** | **Pre-Se** | **MEL** | **Pre-MEL** | **SeNPs** | **Pre-SeNPs** | **MSeNPs**  **(5 mg/kg)** | **MSeNPs**  **(10 mg/kg)** | **Pre- MSeNPs (10 mg/kg)** |
| --- | --- | --- | --- | --- | --- | --- | --- | --- | --- | --- |
| **Viability** | 957.7±1.45 | 614.3±2.3^a,c^ | 532.7±7.51^a,b^ | 360.3±3.48^a,c^ | 211.7±1.2^a,b^ | 452.7±21.36^a,c^ | 370.3±15.34^a,b^ | 281.3±1.45^a,c^ | 253.7±1.45^a^ | 123±12.17^a,b^ |

Different letters indicate significant differences at p < 0.0001. a: all groups vs EAC bearing mice control group; b: pre-treatment groups vs its related treatment ones; c: treatment groups vs MSeNPs at dose 10 mg/kg.

**Table S(3)** Effect of MSeNPs on antioxidants in EAC-bearing mice in different groups

| **Group**  **Parameter** | **EAC** | **Se** | **Pre-Se** | **MEL** | **Pre-MEL** | **SeNPs** | **Pre-SeNPs** | **MSeNPs**  **(5 mg/kg)** | **MSeNPs**  **(10 mg/kg)** | **Pre- MSeNPs (10 mg/kg)** |
| --- | --- | --- | --- | --- | --- | --- | --- | --- | --- | --- |
| **SOD**  **(U/g tissue)** | 312.6±0.88 | 1404±2.05^a,c^ | 1661±29.57^a,b^ | 1836±12.11^a,c^ | 2284±46.15^a,b^ | 2068±4.77^a,c^ | 2840±24.57^a,b^ | 2834±11.6^a,c^ | 4348±70.68^a^ | 6500±3.78^a,b^ |
| **Catalase**  **(KU/g tissue)** | 127.9±0.83 | 456.7±20.48^a,c^ | 769.5±8.11^a,b^ | 543.4±20.29^a,c^ | 845.5±13.59^a,b^ | 544±16.94^a,c^ | 727±8.89^a,b^ | 828.3±29.93^a,c^ | 860.9±11.93^a^ | 1300±89.46^a,b^ |
| **GPX**  **(U/g tissue)** | 2.42±0.11 | 5.45±0.15^a,c^ | 15.11±0.067^a,b^ | 6.05±0.06^a,c^ | 20.95±0.34^a,b^ | 6.45±0.09^a,c^ | 23.1±0.14^a,b^ | 9.2±0.07^a,c^ | 11.42±0.16^a^ | 28.13±0.08^a,b^ |
| **NO**  **(µg/dl)** | 121.2±0.37 | 14.88±0.3^a,c^ | 11.48±0.5^a,b^ | 20.75±0.63^a,c^ | 18.4±0.31^a,b^ | 51.55±0.86^a,c^ | 42.18±0.19^a,b^ | 44.98±0.06^a,c^ | 26.33±0.19^a^ | 11.2±0.23^a,b^ |
| **MDA**  **(nmol/ml)** | 61.4±0.18 | 12.48±0.14^a,c^ | 5.67±0.39^a,b^ | 11.72±0.27^a,c^ | 4.77±0.19^a,b^ | 11.11±0.06^a,c^ | 4.36±0.22^a,b^ | 8.52±0.17^a,c^ | 6.35±0.14^a^ | 4.17±0.08^a,b^ |

## **Table S(4)** Effect of MSeNPs on IL-6 in EAC-bearing mice in different groups

| **Group**  **Parameter** | **EAC** | **Se** | **Pre-Se** | **MEL** | **Pre-MEL** | **SeNPs** | **Pre-SeNPs** | **MSeNPs**  **(5 mg/kg)** | **MSeNPs**  **(10 mg/kg)** | **Pre- MSeNPs (10 mg/kg)** |
| --- | --- | --- | --- | --- | --- | --- | --- | --- | --- | --- |
| **IL-6**  **(pg/mg)** | 505.7±2.25 | 304.6±17.36^a,c^ | 170.4±3.62^a,b^ | 226.9±4.82^a,c^ | 144.3±3.38^a,b^ | 104.3±2.06^a,c^ | 56.05±1.07^a,b^ | 84.52±0.62^a,c^ | 68.65±0.44^a^ | 40.59±2.17^a,b^ |

**Table S(5)** Cell cycle distribution of tumor cells across the different animal groups.

| **Group**  **Parameter** | **EAC** | **Se** | **Pre-Se** | **MEL** | **Pre-MEL** | **SeNPs** | **Pre-SeNPs** | **MSeNPs**  **(5 mg/kg)** | **MSeNPs**  **(10 mg/kg)** | **Pre-MSeNPs**  **(10 mg/kg)** |
| --- | --- | --- | --- | --- | --- | --- | --- | --- | --- | --- |
| **G0/G1**  **(%)** | 39.75±0.52 | 52±0.74^a,c^ | 55.2±0.19^a,b^ | 67.15±0.75^a,c^ | 70.33±1.03^a,b^ | 55.98±0.2.38^a,c^ | 67.35±0.402^a,b^ | 66.05±2.75^a,c^ | 70.31±1.85^a^ | 78.65±2.67^a,b^ |
| **S Phase**  **(%)** | 16.45±0.23 | 8.66±0.067^a,c^ | 23.93±0.042^a,b^ | 5.13±0.021 ^a,c^ | 1.55±0.034^a,b^ | 8.45±0.13 ^a,c^ | 3.2±0.097^a,b^ | 6.33±0.15 ^a,c^ | 1.65±0.15 ^a^ | 1.85±0.23 ^a,b^ |
| **G2/M**  **(%)** | 37.16±0.29 | 27.61±0.18^a,c^ | 6.6±0.36^a,b^ | 6.15±0.37 ^a,c^ | 9.6±0.57^a,b^ | 2.08±0.34 ^a,c^ | 6.78±0.26^a,b^ | 2.61±0.32 ^a,c^ | 8.7±0.23 ^a^ | 3.16±0.15 ^a,b^ |

**
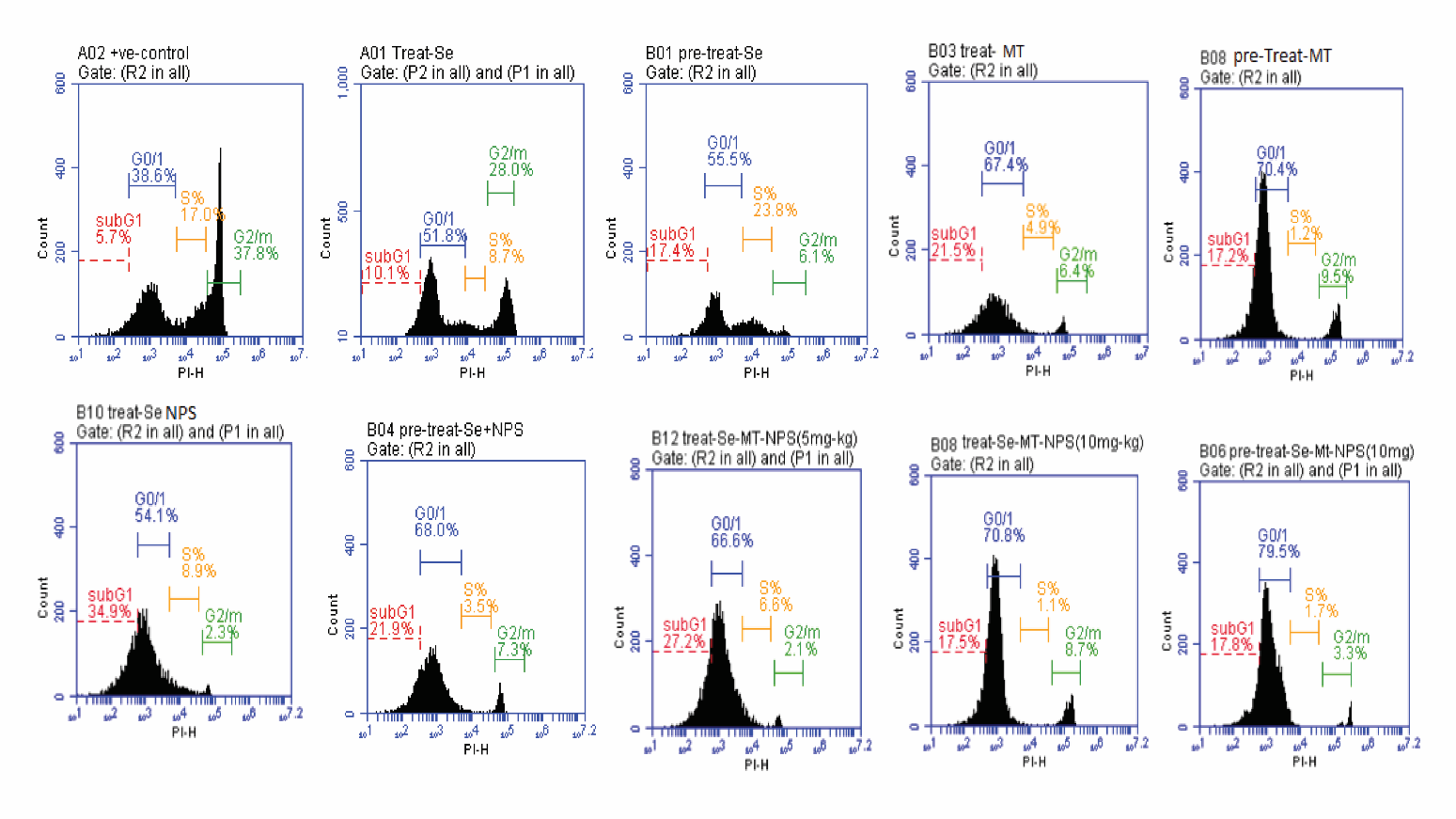
**

**Figure S(1)** Histograms showing cell cycle distribution of Ehrlich Ascites Carcinoma cells: control vs. various treatment groups.

**Table S(6)** Expression of Caspase-3 in tumor cells across the different animal groups

| **Group**  **Parameter** | **EAC** | **Se** | **Pre-Se** | **MEL** | **Pre-MEL** | **SeNPs** | **Pre-SeNPs** | **MSeNPs**  **(5 mg/kg)** | **MSeNPs**  **(10 mg/kg)** | **Pre-MSeNPs**  **(10 mg/kg)** |
| --- | --- | --- | --- | --- | --- | --- | --- | --- | --- | --- |
| **-ve caspase (%)** | 90.93±1.11 | 71.73±1.4^a,c^ | 68.81±0.79^a,b^ | 47.43±0.59^a,c^ | 37.86±1.3^a,b^ | 47.41±1.93^a,c^ | 38.8±1.21^a,b^ | 36.83±1.31^a,c^ | 31.1±0.26^a^ | 23.25±0.83^a,b^ |
| **+ve caspase (%)** | 9.06±1.11 | 28.26±1.4^a,c^ | 31.18±0.79^a,b^ | 52.56±0.59^a,c^ | 62.13±1.3^a,b^ | 52.58±1.93^a,c^ | 61.2±1.21^a,b^ | 63.16±1.31^a,c^ | 68.93±0.26^a^ | 76.75±0.83^a,b^ |


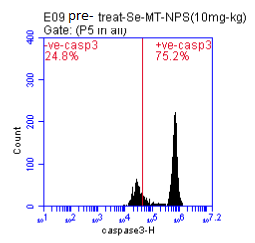


## **Figure S(2)** Histograms of caspase-3 in tumor cells across the different studied animal groups. Caspase-Negative (-ve) Cells indicate viable (non-apoptotic) cells, while Caspase-Positive (+ve) Cells indicate apoptotic cells.

**Table S(7)** Measurement of Ki-67 in the Ehrlich Ascites tumor cells.

| **Group**  **Parameter** | **EAC** | **Se** | **Pre-Se** | **MEL** | **Pre-MEL** | **SeNPs** | **Pre-SeNPs** | **MSeNPs**  **(5 mg/kg)** | **MSeNPs**  **(10 mg/kg)** | **Pre-MSeNPs (10 mg/kg)** |
| --- | --- | --- | --- | --- | --- | --- | --- | --- | --- | --- |
| **Ki-67 (%)** | 90.83±1.53 | 55.83±2.38^a,c^ | 43.33±1.05^a,b^ | 46.66±2.1^a,c^ | 33.33±2.47^a,b^ | 43.33±1.66^a,c^ | 28.33±1.66^a,b^ | 37.83±1.3^a,c^ | 25.83±2^a^ | 17.5±1.11^a,b^ |
